# Modelling distributions of *Aedes aegypti* and *Aedes albopictus* using climate, host density and interspecies competition

**DOI:** 10.1371/journal.pntd.0009063

**Published:** 2021-03-25

**Authors:** Bingyi Yang, Brooke A. Borgert, Barry W. Alto, Carl K. Boohene, Joe Brew, Kelly Deutsch, James T. DeValerio, Rhoel R. Dinglasan, Daniel Dixon, Joseph M. Faella, Sandra L. Fisher-Grainger, Gregory E. Glass, Reginald Hayes, David F. Hoel, Austin Horton, Agne Janusauskaite, Bill Kellner, Moritz U. G. Kraemer, Keira J. Lucas, Johana Medina, Rachel Morreale, William Petrie, Robert C. Reiner, Michael T. Riles, Henrik Salje, David L. Smith, John P. Smith, Amy Solis, Jason Stuck, Chalmers Vasquez, Katie F. Williams, Rui-De Xue, Derek A. T. Cummings

**Affiliations:** 1 Department of Biology, University of Florida, Gainesville, Florida, United States of America; 2 Emerging Pathogens Institute, University of Florida, Gainesville, Florida, United States of America; 3 Department of Entomology and Nematology, Florida Medical Entomology Laboratory, University of Florida, Vero Beach, Florida, United States of America; 4 Polk County Mosquito Control, Parks and Natural Resources Division, Florida, United States of America; 5 Institut de Salut Global de Barcelona, Carrer del Rosselló, Barcelona, Catalonia, Spain; 6 Orange County Government, Florida, Orange County Mosquito Control Division, Florida, United States of America; 7 University of Florida Institute of Food and Agricultural Sciences, Bradford County Extension, Starke, Florida, United States of America; 8 Department of Infectious Diseases and Immunology, University of Florida, Gainesville, Florida, United States of America; 9 Anastasia Mosquito Control District, St. Augustine, Florida, United States of America; 10 Brevard County Mosquito Control, Florida, United States of America; 11 Hernando County Mosquito Control, Florida, United States of America; 12 Department of Geography, University of Florida, Gainesville, Florida, United States of America; 13 Palm Beach County Mosquito Control, Florida, United States of America; 14 Lee County Mosquito Control District, Florida, United States of America; 15 Gulf County Mosquito Control, Florida, United States of America; 16 Pasco County Mosquito Control District, Florida, United States of America; 17 Citrus County Mosquito Control District, Florida, United States of America; 18 Harvard Medical School, Boston, Massachusetts, United States of America; 19 Computational Epidemiology Lab, Boston Children’s Hospital, Boston, Massachusetts, United States of America; 20 Department of Zoology, University of Oxford, Oxford, United Kingdom; 21 Collier Mosquito Control District, Naples, Florida, United States of America; 22 Miami-Dade County Mosquito Control, Florida, United States of America; 23 Institute for Health Metrics and Evaluation, University of Washington, Seattle, Washington, United States of America; 24 Beach Mosquito Control District, Florida, United States of America; 25 Mathematical Modelling Unit, Institut Pasteur, Paris, France; 26 Florida State University, Panama City, Florida, United States of America; 27 Clarke: Aquatic and Mosquito Control Services and Products, St. Charles, Illinois, United States of America; 28 Pinellas County Mosquito Control, Stormwater and Vegetation Division, Florida, United States of America; 29 Manatee County Mosquito Control District, Florida, United States of America; University of California, Davis, UNITED STATES

## Abstract

Florida faces the challenge of repeated introduction and autochthonous transmission of arboviruses transmitted by *Aedes aegypti* and *Aedes albopictus*. Empirically-based predictive models of the spatial distribution of these species would aid surveillance and vector control efforts. To predict the occurrence and abundance of these species, we fit a mixed-effects zero-inflated negative binomial regression to a mosquito surveillance dataset with records from more than 200,000 trap days, representative of 53% of the land area and ranging from 2004 to 2018 in Florida. We found an asymmetrical competitive interaction between adult populations of *Aedes aegypti* and *Aedes albopictus* for the sampled sites. Wind speed was negatively associated with the occurrence and abundance of both vectors. Our model predictions show high accuracy (72.9% to 94.5%) in validation tests leaving out a random 10% subset of sites and data since 2017, suggesting a potential for predicting the distribution of the two *Aedes* vectors.

## Author summary

*Aedes aegypti* and *Aedes albopictus* are two prime mosquito vectors that transmit emerging arboviral pathogens (e.g. dengue virus, Zika virus and chikungunya virus), which cause substantial disease burden in humans. This study attempts to improve previous studies to map the distribution of *Ae*. *aegypti* and *Ae*. *albopictus* with greater validation and provide a finer resolution of current and future projections of mosquito populations. We found evidence of an asymmetrical competitive interaction between *Aedes* vectors where *Aedes aegypti* is suppressed by *Aedes albopictus*. In addition to the role of species interactions, abiotic factors, including meteorological factors and human population density, are important predictors of the distribution of these two *Aedes* mosquito species. Our models demonstrate the potential to predict the occurrence and abundance of the two *Aedes* vectors, which can enhance domestic mosquito control efforts.

## Introduction

*Aedes* mosquitoes, in particular, *Aedes aegypti* (Linnaeus) and *Aedes albopictus* (Skuse), are the primary vectors of multiple arboviruses including dengue virus (DENV), Zika virus (ZIKV), yellow fever virus, and chikungunya virus (CHIKV)[1–3]. The incidence of these viruses in humans is driven, in part, by the close overlapping habitats of humans and these vectors [4]. In the absence of effective vaccines, reducing contact between mosquitoes and humans through targeted mosquito control is regarded as the most effective approach to reducing the risk of mosquito-borne arbovirus transmission. There have been several efforts to create large-scale estimates of the spatial presence and abundance of these vectors using a variety of collection methods and data from literature reports and entomological surveys of mosquito occurrence [5,6]. Global maps have been generated using climate and socio-economic variables, relying on a strong dependence of mosquito populations to temperature and rainfall [7–9]. These efforts have uncertainty associated with publication bias and variability of collection methods. Large-scale data collected by standardized surveillance methods could improve the certainty and precision of occurrence and abundance maps.

Florida has suffered from the introduction and autochthonous transmission of DENV [10,11], CHIKV [12] and ZIKV [13,14] and remains at high risk of transmission due to repeated pathogen introductions, high densities of *Ae*. *aegypti* and *Ae*. *albopictus* [7] and favourable meteorological conditions [13,15]. Studies have shown a positive relationship between human Zika and dengue cases and larger *Ae*. *aegypti* populations in urban areas [13,16]. Therefore, characterizing the population size of the two *Aedes* species over time and space could aid in examining the risk of local arbovirus transmission and spread in Florida and inform more effective and targeted mosquito control efforts.

Although coexistence of the two *Aedes* vectors is reported [17], declining populations and displaced habitats of *Ae*. *aegypti* have been observed in several places, including Florida [3,18–20]. In particular, the habitats of *Ae*. *aegypti* were restricted to urban areas while those of *Ae*. *albopictus* were found to increase in suburban and rural areas in Florida [21]. The proposed mechanisms for the displacement of *Ae*. *aegypti* include species interactions such as the superiority of *Ae*. *albopictus* to compete for resources at the larval stage and asymmetric sterilization at the adult stage after interspecific mating, which favours *Ae*. *albopictus* [1,3,22]. Previous studies modelled the current spatial distribution of *Ae*. *aegypti* and *Ae*. *albopictus* by applying boosted regression trees to a comprehensive global database of *Aedes* occurrence [5,7] and characterized the spatial and temporal abundance of the two *Aedes* species in a local southern Florida county [23–28]. In this study, we build on these previous findings by incorporating longitudinal data collected from a standardized format, providing information on both occurrence and absence with the temporal component. Additionally, a recent systematic review [29] reported inconsistent findings on the associations between the species interactions between *Ae*. *aegypti* and *Ae*. *albopictus* and meteorological factors, we therefore consider these factors in our model.

The objective of this study was to simultaneously characterize the occurrence and abundance of the *Ae*. *aegypti* and *Ae*. *albopictus* mosquitoes using routine mosquito surveillance data in Florida. To estimate if mosquitoes were present or not and, if present, the number of adults in each trap location, a mixed-effects zero-inflated negative binomial (ZINB) regression was performed. Various predictors were examined, like climate and human population density covariates, and their potential impact on *Ae*. *aegypti* and *Ae*. *albopictus* spatial and temporal abundance. In order to evaluate to what extent the model can provide accurate predictions we assessed the performance of the models by validating them against independent data withheld from the model fitting process, especially without the benefits of pre-existing knowledge on abundance and localized spatial variations.

## Methods

### Mosquito surveillance data

Statewide surveillance data on 16 *Aedes* species were obtained by networking with Florida’s mosquito control districts, Clarke Scientific, the Florida Department of Agriculture Consumer Services, and the Florida Department of Health. Each control district is required to trap mosquitoes prior to conducting their control efforts by Florida Statutes 388 and 482. The traps were placed to acquire a representative sampling of the district including baseline traps placed in the same location annually, at risk areas due to environmental factors like increased standing water, locations within areas of known arbovirus transmission, and frequent areas of complaint. Information collected from these traps includes the species-identified count of the trapped adult mosquitoes (total number were calculated where male and female were recorded seperately), date and duration of collection, type of trap (i.e. BG-Sentinels, light traps and other types; details in S1 Table) used, and coordinates of the trap sites. The collected mosquitoes were identified to species level according to standardized mosquito keys [30]. For missing data, the duration of collection was assumed to be one day, according to the common trapping practices, and coordinates were extracted from Google Maps based on the address of the site. The full dataset was aggregated to include data on adult *Ae*. *aegypti* and *Ae*. *albopictus*, two vectors that transmit arboviruses, on a weekly basis. The longitudinal training dataset for the ZINB regression model was extracted from the full dataset and included only data collected from sites with at least four consecutive weeks of surveillance and no missing explanatory variables (S1 Fig).

### Abiotic variables

To examine the potential effects of meteorological factors on the trap rate of the two *Aedes* species, temperature (°C), wind speed (meter per second) and relative humidity (%) were included in the model. We obtained the daily meteorological data for Florida from the regional data of NASA Prediction of Worldwide Energy Resources [31] and applied the inverse distance weighting method [32] to interpolate the daily weather raster of Florida with a 5 km×5 km resolution. To examine the effect of data source, interpolation and spatial resolution on our results, we also conducted sensitivity analyses using meteorological data from National Oceanic and Atmospheric Administration (NOAA, 5 km×5 km) and Daily Surface Weather and Climatological Summaries (Daymet, 1 km×1 km) [33,34]. Data from NOAA was available for stations that were disproportionately distributed in densely populated places, and we interpolated these data using the above-mentioned inverse distance weighting. We downloaded the Daymet data using the coordinates and “daymetr” package [35] without interpolation. The weekly average of weather conditions was calculated as the mean of the weather conditions on the days the traps were collected. To account for the collinearity of the maximum and minimum temperature, we used the residuals of the linear regression of maximum temperature on minimum temperature as a proxy of the maximum temperature in the model, which was calculated as *ΔT_max_* = *T_max_*–(*α*+*βT_min_*), where *T_max_* and *T_min_* denoting the observed maximum and minimum temperature, respectively, while *α* and *β* were estimated from the linear regression. We used human population density as a proxy for urbanization. We used data on population density obtained from the Center for International Earth Science Information Network with a 5 km×5 km resolution for the year 2015 [36]. If the value was missing for a site, we extracted the corresponding environmental variables based on its coordinate and used the average drawn from a 5km buffer around the site.

### Statistical methods

We applied a ZINB regression model to the weekly abundance of *Ae*. *aegypti* and *Ae*. *albopictus* from the longitudinal training dataset, respectively, to account for the excessive zeros in the abundance data and the over-dispersed count of trapped mosquitoes, simultaneously. The ZINB model comprises a binary component (corresponding to the absence/presence of mosquitoes), and a negative binomial component (corresponding to the abundance of mosquitoes). The estimates from the binary component (presented as odds ratio, OR) and the negative binomial component (presented as incidence rate ratio, IRR) represent the associations between the covariates and the occurrence and abundance of these *Aedes* vectors, respectively. The potential factors included in the ZINB model for both species are: the previous abundance of *Ae*. *aegypti* and *Ae*. *albopictus* up to three weeks prior (in log-scale), weekly site-specific meteorological factors (i.e. wind speed, minimum temperature, the residual of maximum and relative humidity), human population density (in log-scale) and type of mosquito traps (i.e. BG-Sentinels, light traps and other types). We examined the potential interaction between *Ae*. *aegypti* and *Ae*. *albopictus* by examining the relationship between the current abundance of one species with the previous abundance of the other species. We used counts of each species detected in recent weeks to predict future weeks. To do this, we only considered records when data was available for four consecutive weeks prior. Trap type was included as an explanatory covariate as each of the traps used has a different effectiveness in trapping each species. We also included the random effects at both site level and county level, which were modelled for both components of the ZINB model simultaneously. The detailed equations used for the ZINB model are provided in the S1 Text. Parameters were estimated by maximizing the likelihood using “glmmTMB” package [37] in R version 3.5.0 (R Foundation for Statistical Computing, Vienna, Austria).

### Goodness of fit of the model

We assessed the goodness of fit of the model by comparing the observations with the predictions of occurrence and abundance from the longitudinal dataset. We assessed the spatial pattern by calculating the site-specific mean of differences between observations and predictions. The absence and presence were assigned as 0 and 1 respectively for calculation purposes. Moran’s I was calculated to assess the spatial autocorrelation of Aedes distribution [38]. We examined the temporal pattern of the model fitting by assessing the monthly 2.5% and 97.5% percentile of the difference between the predicted and observed abundances for the two *Aedes* species.

### Cross-validations

We also performed cross-validation of the model both spatially and temporally. Prediction of a test set was based on a model fit from a training set and comparing the predicted and observed occurrence and abundance. In the spatial prediction, we randomly selected records from 127 (around 10% of total) sites to be the spatial testing set and used the records from the remainder of the sites as a spatial validation training set (S1 Fig). In the temporal prediction, we used data up to the year of 2017 as the temporal validation training set to predict data after 2017 (S1 Fig). The area under the receiver operating characteristic (AUC) was used to measure the performance of prediction on the mosquito occurrence, using the predicted probability of the occurrence as the predictor. In order to assess the accuracy of predictions on abundance, we divided the observed and predicted trap rate (*r*, per trap day) into four categories, i.e. *r* = 0, 0<*r*<10, 10≤*r*<100 and *r*≥100. We define the predictions on abundance as correct if the predicted abundance category is the same with observed abundance category. The proportion of correct predictions were calculated by dividing the number of correct predictions on abundance by the number of traps where the mosquito was found and predicted to occur (excluding the impact of misclassifications of presence/absence).

### Utility of model prediction

In order to assess the model utility in predictions, we first compared the observations of occurrence and abundance from the longitudinal dataset with predictions using models that incorporate 1) both random effects and prior abundance information, 2) random effects only (“no abundance model”), 3) prior abundance information only and 4) none of random effects and prior abundance information. All models accounted for climate factors, human population density and trap types. We evaluated whether the proportion of the predicted presence and abundance categories were consistent with observations. In addition, to test the model’s utility for real-time prediction, we used the “no abundance model” fit using longitudinal data to predict an external “no abundance testing dataset” (S1 Fig) and compared the predictions with observations. The no abundance testing dataset consists of the surveillance records in the full dataset that failed on the four consecutive four-week criteria (S1 and S2 Figs). We also assessed the performance of predictions calculated by removing the random effects estimates of the “no abundance model” in order to illustrate the mean trap rate in Florida.

## Results

**Fig 1:**
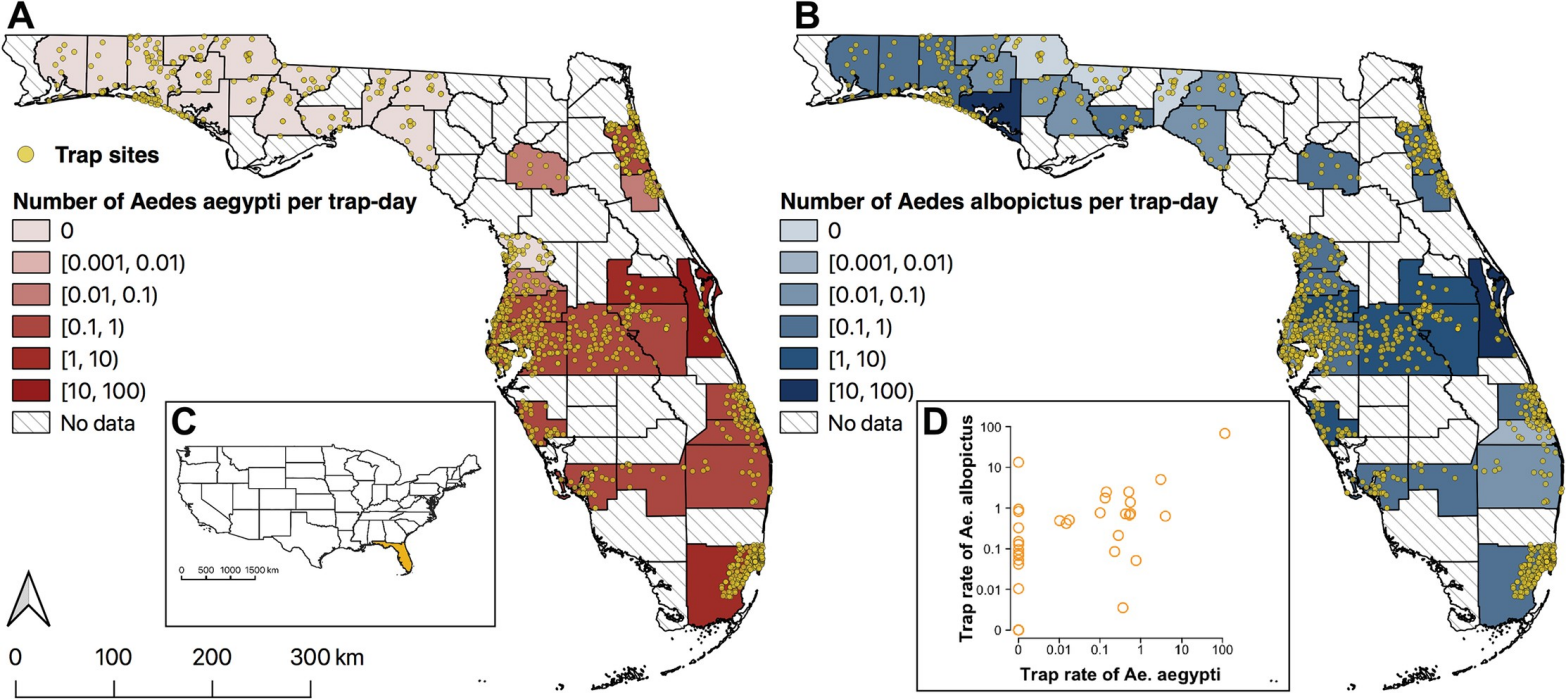
Locations of traps and geographic variation in abundance of *Aedes aegypti* (A) and *Aedes albopictus* (B) in Florida. Color (red for *Ae*. *aegypti*, blue for *Ae*. *albopictus*) indicates mean abundance per trap day in each county. Diagonal lines indicate counties without data. Inset (C) shows the location of Florida (orange) in the contiguous US. Plot (D) shows *Ae*. *aegypti* versus *Ae*. *albopictus* abundances in each county. Maps produced using QGIS Version 3.0.2 (QGIS Development Team, 2018). Source of shapefile: Southwest Florida Water Management District (https://geodata.myflorida.com/datasets/swfwmd::florida-counties).

We integrated a full dataset from counties in Florida that contains 180,242 weekly records and representative of around 102,000 km^2^ (73% of Florida) between 2004 and 2018 (S1 and S3 Figs). From the full dataset, we extracted a longitudinal training dataset that only included data collected from sites where at least four consecutive weeks of surveillance were available. The longitudinal training dataset included 132,088 weekly records from 1,246 unique sites for *Ae*. *aegypti* and *Ae*. *albopictus*, covering 33 out of 67 counties from 2004 to 2018. The land area covered by the counties that we have data for represents 53% of the land area in Florida (Table 1 and S1 and S3 Figs and S1 Video). Traps were typically set for one day but a minority of collaborators reported counts from a trap that was set for multiple days (7.4%). Approximately 87.4% and 84.8% of trap episodes reported no adults collected for *Ae*. *aegypti* or *Ae*. *albopictus*, respectively. The majority (81.4%) of traps used were light traps, and the remaining 7.3% and 11.3% of traps used were BG Sentinel traps or other mosquito traps (Tables 1 and S1), respectively. A wider distribution range and higher trap rate was reported for *Ae*. *albopictus* compared to *Ae*. *aegypti* in Florida, and, as expected from previous studies, most *Ae*. *aegypti* were reported in central and southern Florida (Figs 1 and S3). Both *Ae*. *aegypti* and *Ae*. *albopictus* were trapped more often between May to October (S3 Fig and S1 Video).

**Table 1:** Characteristics of surveillance of *Aedes aegypti* and *Aedes albopictus* in Florida, 2004–2018.

Characteristic	Number (%)	
	Longitudinal training dataset	No abundance testing dataset
**Number of Counties**	33	48
**Number of Sites**	1,246	2,791
**Number of Trap-days**	235,677	57,469
**Records**	132,088	45,535
*Aedes aegypti*		
Absence	115,447 (87.4%)	39,384 (86.5%)
Presence	16,641 (12.6%)	6,151 (13.5%)
*Aedes albopictus*		
Absence	112,021 (84.8%)	35,667 (78.3%)
Presence	20,067 (15.2%)	9,868 (21.7%)
**Trap Types**		
Light trap	107,571 (81.4%)	31,176 (68.5%)
BG Sentinel	9,518 (7.2%)	5,648 (12.4%)
Other trap types	14,999 (11.4%)	8,711 (19.13%)

The median human population density at the locations where the traps were set is 480.8 persons per km^2^ (Interquartile range (IQR), 112.5 to 1,165.2 km^2^) (S4 and S5 Figs). The median weekly average wind speed was 5.4 meters per second (IQR, 4.5 to 6.6 m/s), and the median relative humidity was 76.7% (IQR, 73.1 to 80.1%) (S4 Fig). The minimum temperature of the time when the trap was set ranged from 18.7 to 25.8°C with a median of 23.0°C.

### Presence and abundance of *Aedes*

The results from ZINB regression suggested the probability of presence of *Ae*. *aegypti* and *Ae*. *albopictus* in the current week was positively associated with their respective abundance in the previous week. (Table 2). The abundance of both *Aedes* species was more likely to be higher if a higher abundance was reported for its own species (e.g. Incidence rate ratio (IRR) 1.03 and 1.02 for one week prior for the two vectors, respectively) (Table 2). The abundance of *Ae*. *aegypti* was negatively associated with the abundance of *Ae*. *albopictus* in the last three weeks (IRR: 0.992, 0.994 and 0.990 for one, two and three weeks earlier, respectively), while the abundance of *Ae*. *albopictus* was not associated with the previous abundance of *Ae*. *aegypti* (Table 2).

**Table 2:** Estimates of odds ratio (OR) and incidence rate ratio (IRR) from mixed-effects zero-inflated negative binomial analysis of *Aedes aegypti* and *Aedes albopictus* in Florida, 2004–2018.

Variables	*Aedes aegypti*		*Aedes albopictus*	
	OR (95% CI^†^)	IRR (95% CI^†^)	OR (95% CI^†^)	IRR (95% CI^†^)
**Previous *Ae*. *aegypti* abundance/presence (per trap-day)**				
Trap rate in week *t-1*	2.46 (2.19, 2.76)*	1.03 (1.02, 1.03)*	1.21 (1.10, 1.34)*	1.00 (1.00, 1.01)
Trap rate in week *t-2*	2.36 (2.11, 2.65)*	1.03 (1.03, 1.03)*	1.42 (1.28, 1.57)*	1.00 (0.99, 1.00)
Trap rate in week *t-3*	1.84 (1.64, 2.07)*	1.02 (1.01, 1.02)*	1.04 (0.94, 1.15)	1.00 (1.00, 1.01)
**Previous *Ae*. *albopictus* abundance/presence (per trap-day)**				
Trap rate in week *t-1*	1.30 (1.16, 1.47)*	0.99 (0.99,1.00)*^†^	2.48 (2.32, 2.65)*	1.02 (1.02, 1.03)*
Trap rate in week *t-2*	1.44 (1.29, 1.62)*	0.99 (0.99,1.00)*^†^	2.19 (2.05, 2.35)*	1.02 (1.01, 1.02)*
Trap rate in week *t-3*	1.28 (1.14, 1.44)*	0.99 (0.99,1.00)*^†^	1.68 (1.57, 1.80)*	1.02 (1.01, 1.02)*
**Human population density (100 *persons per km*^2^)**	1.05 (1.03, 1.07)*	1.00 (0.99, 1.02)	0.95 (0.94, 0.97)*	0.98 (0.97,1.00)*^†^
**Meteorology**				
Average wind speed (*m/s*)	0.98 (0.95, 1.01)	0.97 (0.96, 0.99)*	0.97 (0.95, 0.99)*	0.97 (0.95, 0.98)*
Minimum temperature (°*C*)	1.01 (0.99, 1.02)	1.13 (1.12, 1.14)*	1.08 (1.07, 1.09)*	1.09 (1.08, 1.10)*
Residual of maximum temperature (°*C*)	1.12 (1.03, 1.21)*	0.91 (0.87, 0.95)*	1.01 (0.95, 1.06)	1.04 (1.00,1.08)*^†^
Relative humidity (%)	1.01 (1.00, 1.02)	0.99 (0.98,1.00)*^†^	0.99 (0.98, 0.99)*	1.00 (0.99, 1.00)
**Trap type**				
BG sentinel	Ref.	Ref.	Ref.	Ref.
Light trap	0.00 (0.00, 0.01)*	0.40 (0.31, 0.51)*	0.77 (0.60,1.00)*^†^	0.29 (0.24, 0.36)*
Other	0.01 (0.00, 0.02)*	0.20 (0.14, 0.29)*	1.77 (1.28, 2.44)*	0.25 (0.19, 0.33)*
**Random effects**				
Site	1.34	1.67	1.40	0.90
County	12.27	2.82	6.59	1.56
**Dispersion parameter**	--	1.46 (1.42, 1.51)	--	1.13 (1.10, 1.17)

* P < 0.05.

^**†**^ Credible interval.

† The values with three effective digits for these estimates are (from right to left by row): 0.992 (0.987, 0.998), 0.994 (0.988, 0.999), 0.990 (0.985, 0.996), 0.984 (0.969, 0.998), 1.041 (1.001, 1.083), 0.986 (0.979, 0.994) and 0.775 (0.600, 0.999).

We found both the presence (Odds ratio (OR): 0.98, 95% confidence interval (CI) 0.95 to 1.01 and 0.97, 95% CI, 0.95 to 0.99, respectively) and abundance (IRR: 0.97, 95% CI, 0.96 to 0.99 and 0.97, 95% CI, 0.95 to 0.98, respectively) of *Ae*. *aegypti* and *Ae*. *albopictus* were negatively associated with the average wind speed of the week, although such association was not significant for the presence of *Ae*. *aegypti*. Minimum temperature was positively associated with the occurrence (OR: 1.08, 95% CI, 1.07 to 1.09) for *Ae*. *albopictus* and the abundance (IRR: 1.13, 95% CI, 1.12 to 1.14 for *Ae*. *aegypti* and 1.09, 95% CI, 1.08 to 1.10 for *Ae*. *albopictus*). Residuals of maximum temperature was found to be negatively associated with the abundance of *Ae*. *aegypti* (OR: 0.91, 95% CI, 0.87 to 0.95) but positively associated with the abundance of *Ae*. *albopictus* (OR: 1.04, 95% CI, 1.00 to 1.08) (Table 2). We found the relative humidity was negatively associated with the abundance of *Ae*. *aegypti* (IRR: 0.99, 95% CI, 0.98 to 1.00) and the occurrence of *Ae*. *albopictus* (IRR: 0.99, 95% CI, 0.98 to 0.99). Model estimates using climate data from alternative sources were similar to our main results, except for the positive associations between maximum temperature and the abundance and presence for both species (S2 and S3 Tables). Greater precipitation was positively associated with the abundance for *Ae*. *aegypti* (IRR: 1.42, 95% CI, 1.26 to 1.59), but not associated with the probability of presence (OR: 0.85, 95% CI, 0.69 to 1.05 for *Ae*. *aegypti* and 1.05, 95% CI, 0.94 to 1.19 for *Ae*. *albopictus*, respectively) (S2 Table).

Both the probability of presence (OR: 0.95, 95% CI, 0.94 to 0.97) and abundance (IRR: 0.98, 95% CI, 0.97 to 1.00) of *Ae*. *albopictus* were negatively associated with a higher human population density, while the probability of the presence of *Ae*. *aegypti* was positively associated with human population density (OR: 1.05, 95% CI, 1.03 to 1.07). The probability of presence and abundance of *Ae*. *agypti* and *Ae*. *albopictus* was lower when using light traps and other trap types, compared to using BG traps (Table 2). We also found substantial heterogeneities of presence and abundance of these two *Aedes* species across trap sites and counties (Table 2). The heterogeneity was greater at the county level (random effects (RE): 12.27 for *Ae*. *aegypti* and 6.59 for *Ae*. *albopictus*) compared to the site level for both species.

### Goodness of fit of the model

**Fig 2:**
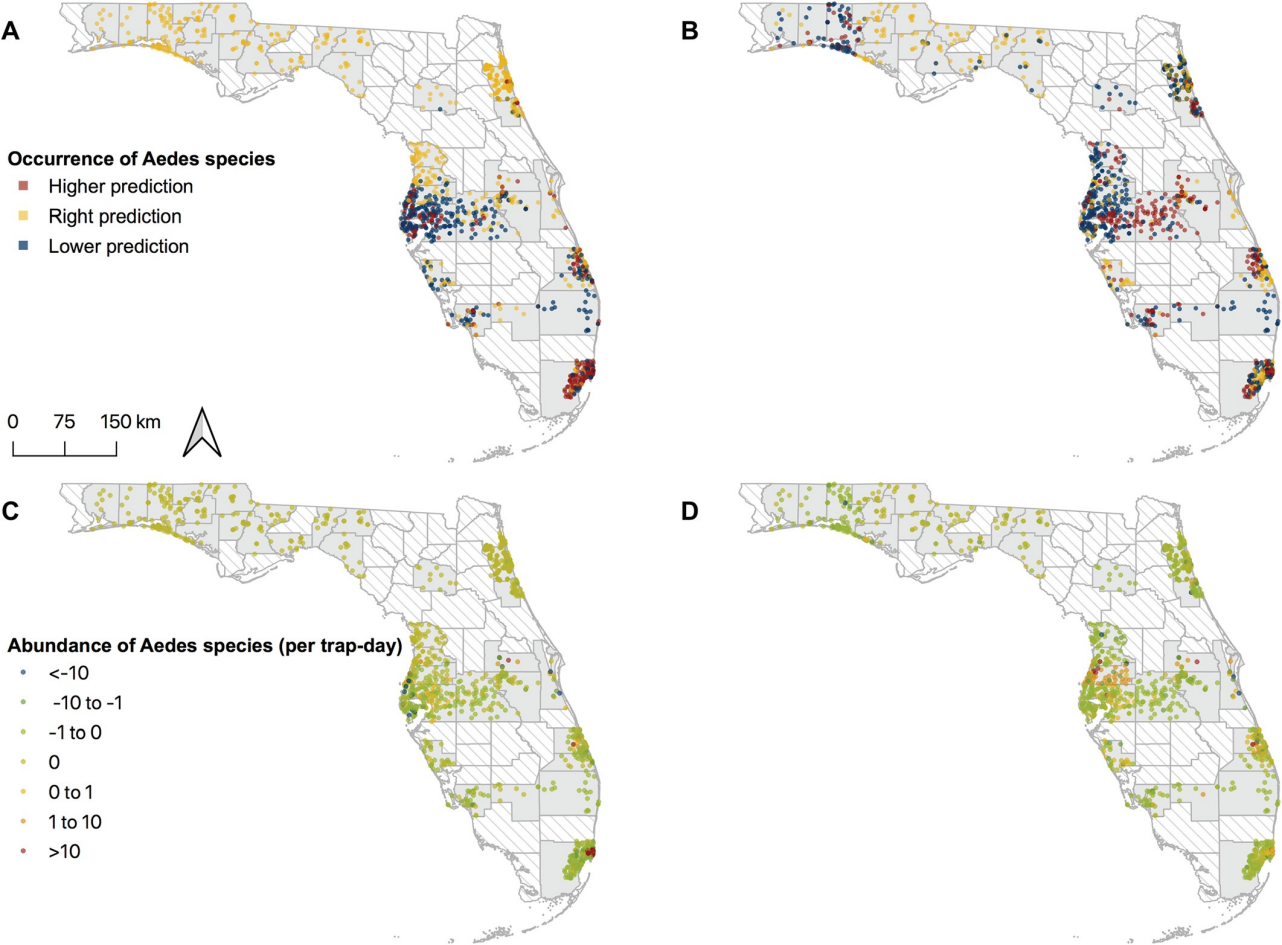
Geographic variations in model predictions in occurrence and abundance of *Aedes aegypti* and *Aedes albopictus*. (A) Differences in occurrence of *Ae*. *aegypti*. (B) Differences in occurrence of *Ae*. *albopictus*. (C) Differences in abundance of *Ae*. *aegypti*. (D) Differences in abundance of *Ae*. *albopictus*. Each trap site may have multiple predictions from different time points, so values presented here are the mean differences between predictions and observations for each trap site. Maps produced using QGIS Version 3.0.2 (QGIS Development Team, 2018). Source of shapefile: Southwest Florida Water Management District (https://geodata.myflorida.com/datasets/swfwmd::florida-counties).

**Fig 3:**
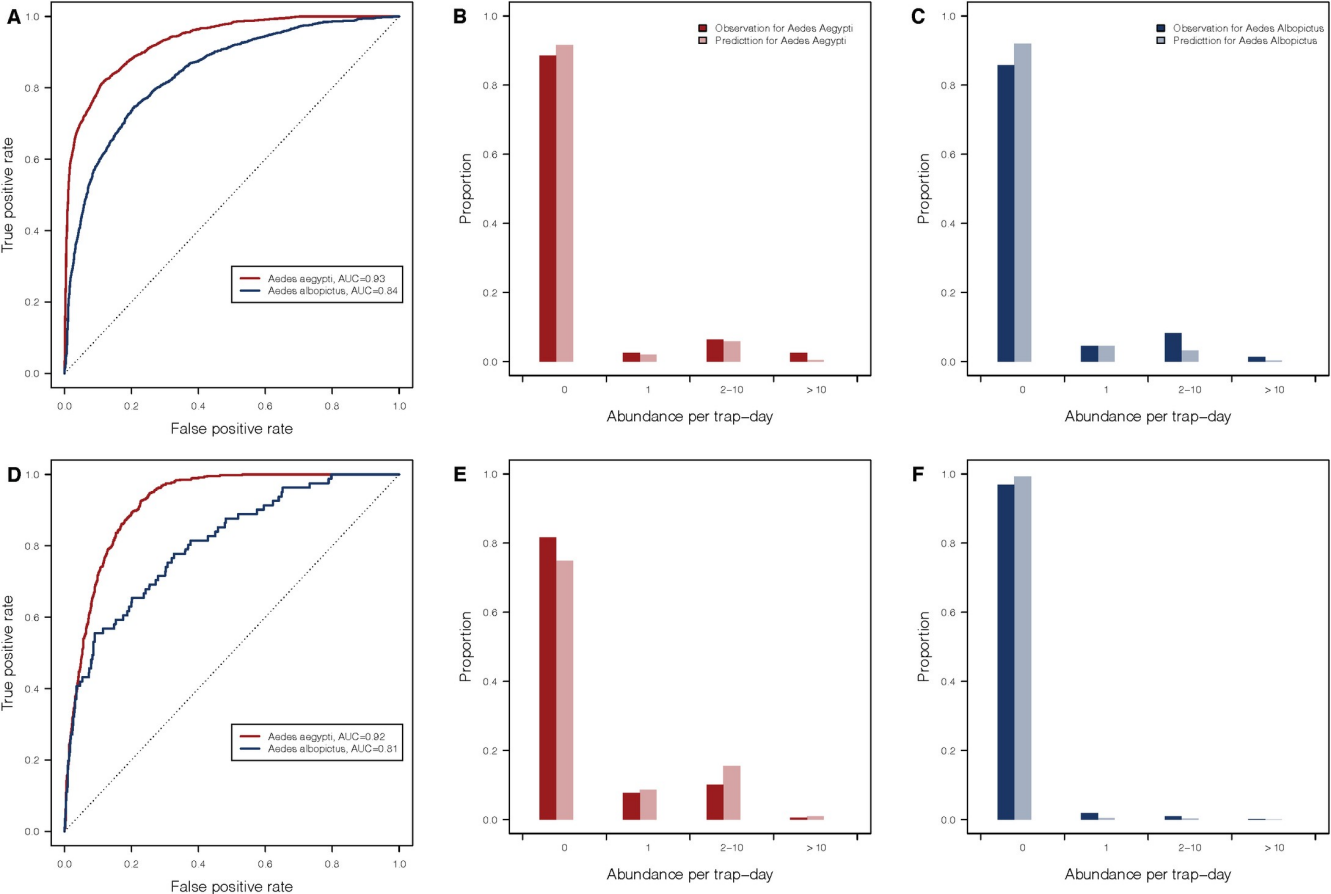
Model performance of predictions in occurrence and abundance of *Aedes aegypti* and *Aedes albopictus* for cross-validations. (A-C) Records from 10% of trap sites were randomly selected as the test set and records from the rest of the traps were the training set. (D-F) Records from 2003 to 2016 were selected as the test set and records during and after 2017 were in the training set. The model was fit to the training set and predicted the test set.

We compared the predictions from the main ZINB model with observed presence and abundance from the longitudinal training datasets (Figs 2 and S6 and S2 Video). Overall, our model fits well with both the occurrence and abundance estimates for *Ae*. *aegypti* and *Ae*. *albopictus* (Figs 2 and 3). Places where inconsistent predictions and observations on presence/absence were observed are places with higher trap rates of the two species (Fig 2). We observed that 91.1% (95% CI, 91.0% to 91.3%) and 84.9% (95% CI, 84.7% to 85.1%) of the predicted presence/absence was consistent with the observations of *Ae*. *aegypti* and *Ae*. *albopictus*, respectively (S4 Table). Similarly, 78.7% (*Ae*. *aegypti*, 95% CI, 77.9% to 79.4%) and 84.9% (*Ae*. *albopictus*, 95% CI, 84.3% to 85.5%) of the predicted abundance was consistent with the observations among traps where the mosquito was captured and predicted to be present (S4 Table). The values of Moran’s I are 0.47 (p < 0.01) and 0.08 (p = 0.02) for *Ae*. *aegypti* and *Ae*. *albopictus*, respectively, and is -0.03 (p = 0.81) for *Ae*. *aegypti* after removing data from Miami-Dade (S5 Table). Temporal differences and average trap rates were relatively higher between May and September for both species (S6 Fig).

### Cross-validations

We performed the cross-validations both spatially (i.e. holding out 10% of sites) and temporally (i.e. holding out data after 2017). For spatial (Fig 3A) and temporal (Fig 3D) cross-validations, the model predictions are highly consistent with the observed presence of both *Ae*. *aegypti* (AUC: 0.93 and 0.92 for spatial and temporal, respectively) and *Ae*. *albopictus* (AUC: 0.85 and 0.76 for spatial and temporal, respectively). Overall, 72.1% (*Ae*. *aegypti*, 95% CI, 69.3% to 74.9%) and 75.3% (*Ae*. *albopictus*, 95% CI, 72.9% to 77.6%) of the predicted abundance are consistent with the observations among traps where the mosquito was captured and predicted to be present for the spatial validation, while the percentages are 91.1% (*Ae*. *aegypti*, 95% CI, 87.9% to 93.7%) and 100% (*Ae*. *albopictus*, 95% CI, 86.8% to 100%) for the temporal validation (S6 Table).

### Utility of model prediction

**Fig 4:**
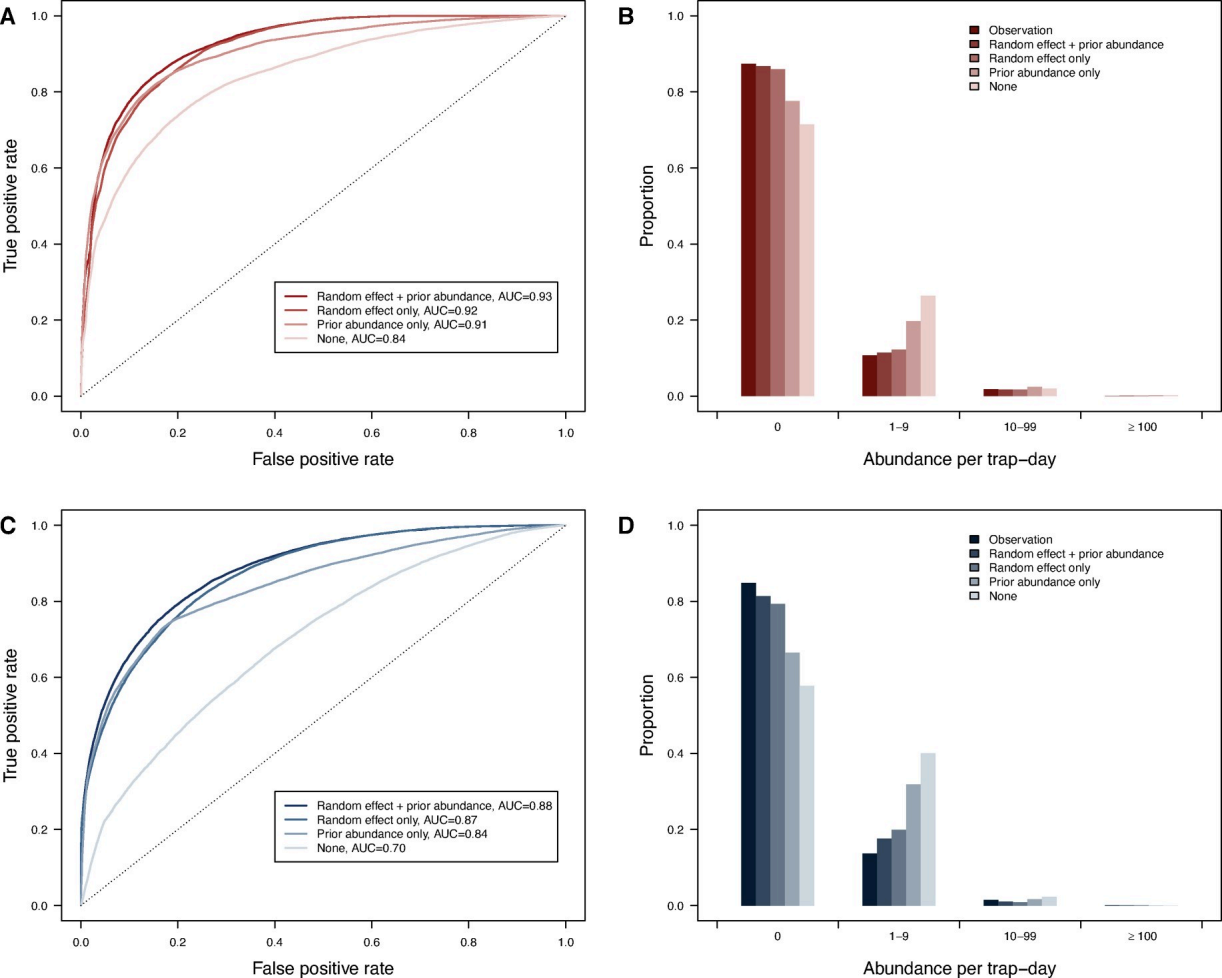
Model performances for different combinations of random effects and prior abundance information.

In order to assess the minimum information needed for the model to provide accurate predictions, we fit the different models incorporating various combinations of random effects and prior abundance information. We found greater reductions in goodness of fit when only random effects were removed than when only removing prior abundance information (Fig 4 and S4 Table). The goodness of fit was reduced by 15.0% (*Ae*. *aegypti*) and 26.1% (*Ae*. *albopictus*) for the model simultaneously when removing random effects and prior abundance compared to the model that included both.

**Fig 5:**
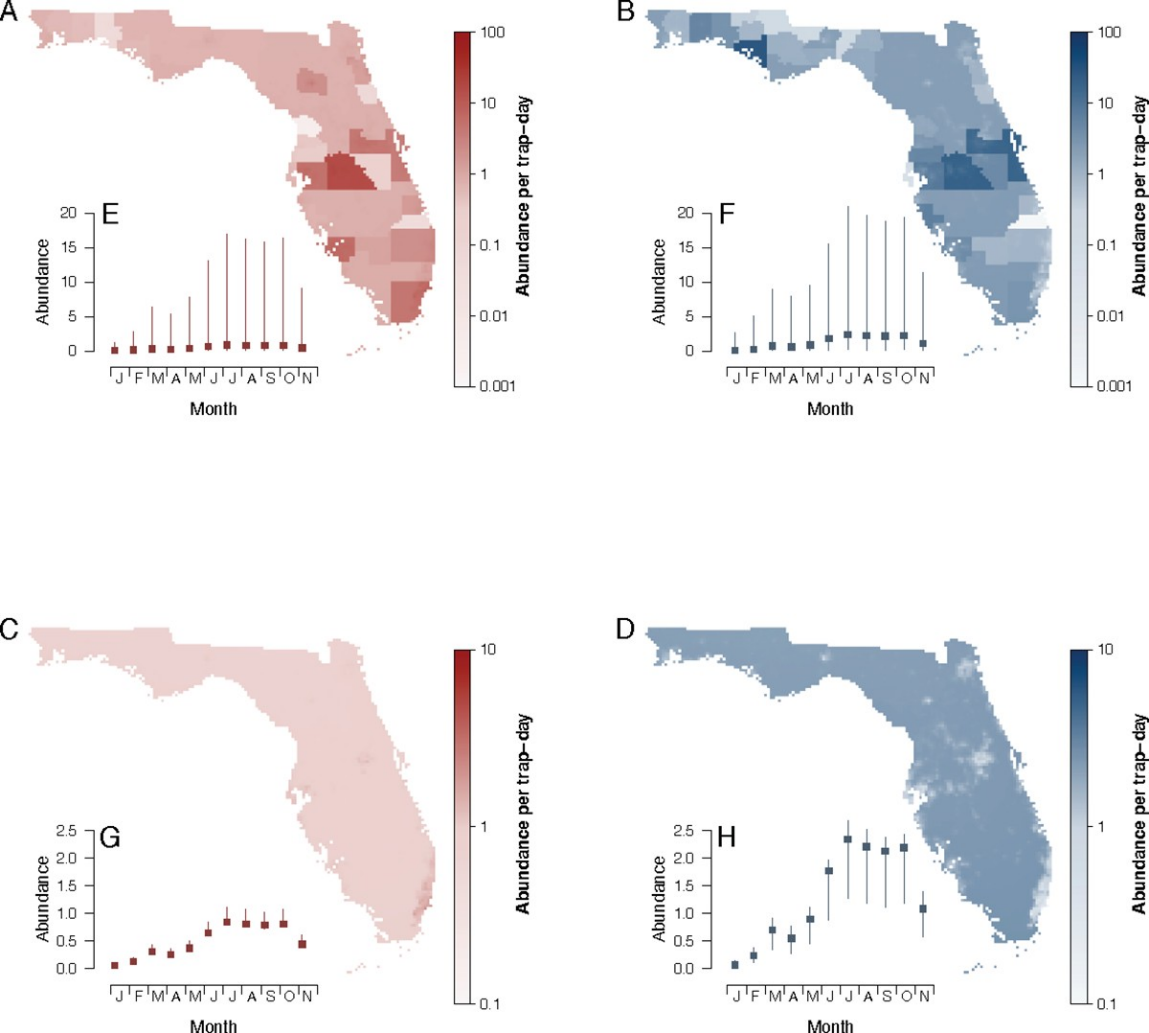
Maps of predicted abundance of *Aedes aegypti* (red, A and C) and *Aedes albopictus* (blue, B and D) on August 1, 2018 in Florida. Predictions are derived from “no abundance model”. Parts A and B show results incorporating random effects which represents differences in trapping counts by county. Parts C and D show results only incorporating fixed effects. Predictions for each month in 2018 are shown in S3 Video. E–H, points and vertical lines are the median and interquartile range of the corresponding predictions across the state at each time point. Maps produced using R Version 3.5.0 (R Foundation for Statistical Computing, Vienna, Austria). Source of shapefile: Southwest Florida Water Management District (https://geodata.myflorida.com/datasets/swfwmd::florida-counties).

Using the model that included random effects but not prior abundance information (“no abundance model” hereafter), we predicted the trap rate of *Ae*. *aegypti* and *Ae*. *albopictus* at all points in the state in 2018 with BG traps (Figs 5 and S7 and S7 Table). Results in Fig 5 show the predictions incorporating random effects and represent systematic differences in trap rates by county (Fig 5A and 5B). The predictions from fixed effects, which represent the mean trap rate, can capture more temporal trends than spatial heterogeneity in Florida (Fig 5C and 5D). We further conducted validation of the above predictions with the no abundance testing dataset (S1 Fig), which is an external dataset that was not used for model training. The performance of model incorporating random effects was better (AUC: 0.90 for *Ae*. *aegypti* and 0.85 for *Ae*. *albopictus*) than predictions of only fixed effects (AUC: 0.81 for *Ae*. *aegypti* and 0.75 for *Ae*. *albopictus*) (S7 Fig and S7 Table).

## Discussion

We built models using more than 132,000 routine mosquito surveillance records from 33 counties in Florida collected from 2004 to 2018 to characterize and predict the occurrence and abundance of *Ae*. *aegypti* and *Ae*. *albopictus*. Our analysis is set apart by the extensive localized data set that was collated from counties throughout Florida, which strengthens the predictive power of our model. Our model performed well, particularly considering the stochastic nature of mosquito populations, trap efficiency and small-scale trap locations. We modelled random effects across sites and counties to account for unmeasured abiotic factors, inconsistencies, and randomness and found the highest random effect was for the probability of presence at the county level, suggesting great heterogeneity of occurrence across counties possibly down to differences in varying micro-scale environment, surveillance, and domestic mosquito control across counties.

Our results suggest a broad distribution of *Ae*. *albopictus* in Florida, while *Ae*. *aegypti* was more likely to be found in counties in southern Florida, a pattern similar to reports during the past two decades [17]. Such results were consistent when using different sources of climate data. This is also consistent with previous observations about the declining population of *Ae*. *aegypti* after the invasion of *Ae*. *albopictus* in the southern United States [21,39]. However, there is some evidence to suggest limited local recoveries of *Ae*. *aegypti* in relation to *Ae*. *albopictus*, in part, attributable to evolution of resistance to satyrization [1,17,40,41]. Our findings on the positive association between the probability of presence of adult mosquitoes of the two *Aedes* species suggest their niches have some overlap, particularly in urban areas [42]. This is supported by the observed coexistence of *Ae*. *aegypti* and *Ae*. *albopictus* in Florida [3,23,39] and the similar breeding behavior of the two species [43]. The abundance of *Ae*. *aegypti* was negatively associated with the previous abundance of *Ae*. *albopictus*, with the greatest effective size observed for the abundance of *Ae*. *albopictus* during the previous three-week period. These results suggest competitive interactions between the two species. A previous study revealed the breeding preference of *Ae*. *aegypti* in habitats without *Ae*. *albopictus* [43]. Our findings support the hypothesis that the two *Aedes* species can coexist but the abundance of adult *Ae*. *aegypti* are suppressed due to its failure to outcompete at the larval stage and/or the impact of interspecific mating [3,22,40]. Evolution of resistance to interspecific mating (i.e., satyrization-resistance) in *Ae*. *aegypti* populations is likely to promote coexistence [1]. Future control efforts targeting the *Aedes* species, especially *Ae*. *albopictus*, need to consider the risk of resurgence of *Ae*. *aegypti*, which has been documented in Brazil [2], and could be possible in Florida considering recent reports of the rapid evolution of satyrization-resistant *aegypti* [1], coupled with an observed increased in insecticide resistance as compared to *Ae*. *albopictus* [44].

We found the presence and abundance of *Ae*. *albopictus* are negatively associated with human population density, while the presence of *Ae*. *aegypti* was positively associated with the human population density, which matches with reports that anthropophilic *Ae*. *aegypti* are more likely to be found in urban areas and *Ae*. *albopictus* has wider range of habitats including peri-urban, vegetated and rural areas [3,45], mostly due to its wide range of host preference and a greater adaptation to different climates [39]. Land cover status, which is an important predictor of distribution of these species by other reports [7,46], was not included in our main analysis as it may be associated with the human population density and the vast majority of the *Ae*. *aegypti* were collected in developed areas with a large human presence, consistent with other studies [47,48]. The observed positive association between human and *Ae*. *aegypti* densities has practical implications for targeted mosquito control because these areas represent the greatest risk for arboviral infections (e.g., dengue [49]).

Elevated abundance of both species between May and October has also been reported. This timeframe corresponds to Florida’s rainy season, associated availability of breeding sites, and a warmer temperature [23]. Related to these abiotic factors, the negative association between wind speed and the presence and abundance of both species can be explained by high wind speed hindering the effective trapping of the mosquitoes [50,51]. Traps are more likely to under-catch mosquitoes on windy days. Likewise, mosquito host-seeking activity has been shown to be impacted by higher wind speeds, which is presumably due to the wind’s influence on flight distance, pattern, and poor dispersal of the CO_2_ plume over both short and long distances.

Furthermore, our results suggest positive associations between the minimum temperature and the observed abundance of adult *Ae*. *aegypti* and *Ae*. *albopictus* when using the NOAA data. However, inconsistent findings on the association between the residuals of maximum temperature and the abundance of *Ae*. *aegypti* was found when using the NASA data. One study suggested higher tolerance of low temperatures in adult *Ae*. *aegypti* compared to *Ae*. *albopictus* leading to a relatively lower mortality of adult *Ae*. *aegypti* at low temperatures and a milder effect of temperature on the presence of *Ae*. *aegypti* [52]. Alternatively, another study observed that *Ae*. *albopictus* prefer to live in cooler areas in Florida [39]. However, different local adaptations by these *Aedes* species to climatic changes were reported both in and out of Florida [3,53].To investigate this, we modeled the residuals of maximum temperature to avoid its collinearity with minimum temperature. This might, however, hinder the interpretation of the residuals of maximum temperature due to the non-linear association between temperature and mosquito survival. Further, the same residuals of maximum temperature may impact the mosquito differently conditional on different minimum temperatures. We performed a sensitivity analysis using mean temperature only, and the associations identified in this study seem to be unaffected by the choice of temperature (S8 Table). Despite these discussions pertaining to the relation between mortality of the two *Aedes* vectors and temperature, seasonality can be used to predict the patterns of presence and abundance of these two *Aedes* species and the incidence of diseases transmitted by the these mosquito vectors [15,23,54].

We find a negative correlation between relative humidity and the abundance of *Ae*. *aegypti* and the presence of *Ae*. *albopictus*. These findings support laboratory and field observations showing climate-driven egg mortality, with greater desiccation resistance in *Ae*. *aegypti* than *Ae*. *albopictus*, and species-specific responses in occupancy of containers with drier conditions favoring *Ae*. *aegypti* [55–57]. Previous field studies have shown that dry periods are associated with disproportionately greater mortality *of Ae*. *albopictus* eggs than *Ae*. *aegypti* eggs in Florida [57]. Previous laboratory studies revealed desiccation stress on survival of adult *Ae*. *aegypti* and *Ae*. *albopictus* with mortality increasing non-linearly with decreasing relative humidity [58–60]. The complex relationship between adult survival, relative humidity, and the observed higher relative humidity in Florida could drive the negative association (S3 Fig). In addition, higher relative humidity was usually associated with greater precipitation, which was found to be positively correlated with the abundance of *Ae*. *aegypti*, but not the probability of occurrence of the two species in the sensitivity analysis (S2 Table). The effect of precipitation on the abundance of these two *Aedes* species was considered to be mediated by induced egg hatching in containers upon flooding and promotion of vegetation after raining [23,29]. The larger effect of precipitation on the abundance of *Ae*. *aegypti* than of *Ae*. *albopictus* could be due to this species’ preference for ovipositing in artificial containers, which are prone to have more obvious influence from precipitation compared to vegetation.

The probability and efficacy of capturing *Ae*. *aegypti* and *Ae*. *albopictus* by a BG-sentinel trap was found to be greater compared to light traps (Table 2), which is consistent with previous findings [61,62]. We performed sensitivity analysis by fitting the model to data collected by BG sentinel traps only or light traps only (S9 Table). We found the robustness of our main results are seemingly unaffected by the spatial distribution of BG sentinel traps (S8 Fig). In addition, we were not able to assess the role of attractants due to limited data available, which are believed to increase the capture efficacy of mosquitoes [63].

Results from the assessment of goodness of fit and cross validation suggested that our model can provide highly accurate predictions on the presence and abundance of *Ae*. *aegypti* and *Ae*. *albopictus*, especially when the model incorporates the previous abundance of heterospecific and conspecific *Aedes* species at a trap. Analysis of long-term mosquito surveillance data is challenged by the excessive zero counts, which may be real absence, or absence due to trap failure or adverse environmental conditions. The ZINB regression can model the two scenarios of absence simultaneously. A larger rate of inaccurate predictions was observed during months when trap rates of both mosquito species were higher, which is due to the more dispersed variance of a higher trap rate and the exponential growth of mosquito populations. The relatively higher inconsistent proportion between observed and predicted occurrence in places with higher trap rates (Fig 2) is caused by the higher chance for false absences in places where *Aedes* can be found. In addition, spatial autocorrelation was found for the model of *Ae*. *aegypti*, which was mainly due to the high autocorrelation between observations in Miami-Dade. The estimates and predictions are however not affected by the spatial autocorrelation, as suggested by the model fit to the longitudinal training dataset removing data from Miami-Dade (S9 Fig and S5 Table).

In order to account for the autocorrelations of the repeated measurements from the same trap and the potential unmeasured confounders that correlated with locations, we incorporated random effects in the model. However, we found a large proportion of spatial variations was explained by the random effects at county level. Despite the fact that the results from the “no abundance model” incorporating fixed effects are only consistent with previous predictions on the two *Aedes* vectors occurrence and survival probability in Florida [8,64,65], the maps are less heterogeneously predictive compared to models incorporating random effects. This is because both 5km × 5km climate and human population density demonstrate relatively less spatial variation in Florida, while the empirical data suggested great variation in the abundance captured across counties (Figs 1 and S1), which could be partially explained by climate data at a finer spatial resolution (as suggest by results using Daymet data). The county-level random effects could be largely mediated by the pre-existing niches of the two *Aedes* vectors, which are the result of unmeasured abiotic factors (S3 Table). Such unmeasured abiotic factors could be the systematic differences in mosquito surveillance across counties or other factors that are critical for mosquito survival, such as the micro-scale climate conditions, the density of mosquitoes favoring micro-environment (e.g. water containers and abandoned tires), the density of hosts other than human, the stochasticity of the establishment of habitat and the varying efforts of vector spraying across counties. Ideally, the model performance could be increased after considering these factors, but it is practically challenging to obtain such information.

Our no abundance model, which used random effect but not the prior abundance information, demonstrated good potential to provide real-time predictions on the occurrence and abundance of *Aedes*, especially in places with long-term mosquito surveillance. Although the performance on predictions (especially for occurrence/presence) for the two *Aedes* vectors could be affected by lack of information on local variations when extrapolating to outside counties, our model can still provide satisfactory predictions. In addition, the performance of temporal validations could be affected by the out-of-sample predictions as many counties started mosquito surveillance after 2017 (S1 and S3 Figs). The dynamic changes of niches of the mosquitoes may further hinder the distribution forecasting of the two vectors. Incorporating the species invasion process could help to improve modeling at places where rich spatial information is available.

Many efforts have been made to map the distribution of *Ae*. *aegypti* and *Ae*. *albopictus* at broad regional scales, which were highly dependent on vegetation and meteorological factors [7–9,64,65]. Places like Florida are theoretically suitable for *Aedes* survival and previous predictions using coarser scale climate and host density may have reduced utility for local mosquito control due to the relatively small spatial variations of such predictors at a fine scale. Results from this study suggested greater spatial variations that cannot be explained by climate factors and host population density alone, which calls for more detailed localized data to further aid predictions at fine scale. In addition, our study observes suppression of adult population of *Ae*. *aegypti* by *Ae*. *albopictus*, highlighting the importance of including species interactions in future mapping work as underscored by recent studies, especially when considering predictions at high spatial resolution [42]. Otherwise, the distribution of *Ae*. *aegypti* would likely be overestimated since the two *Aedes* vectors shared many common abiotic conditions. In addition, integrating standardized longitudinal mosquito surveillance could provide valuable information on absence and abundance, therefore reducing the sampling bias and disproportional weighting caused by presence only data [66]. By creating and making such a dataset available, it also enables the temporal predictions on presence and abundance, rather than a single prediction on occurrence.

There are several limitations to our study. First, our data has relatively more trap episodes during April to November, when the trap rate for these two vectors was often high. The estimated impact of low temperature on the presence and abundance of these two *Aedes* vectors may therefore be affected. Second, more than half of the records included in the main analysis are from Miami-Dade, St. Johns, Polk and Pinellas counties (S10 Table). We have modelled the random effects across both sites and counties to account for the potential spatial variations of surveillance, which may improve the generalization capability of our conclusions. We were not able to characterize specific details of trap locations or other aspects of the built environment, such as the micro-scale climate and environment. These details could explain more spatial variations in the distribution of the mosquitoes, as suggested by our sensitivity analysis that used a finer spatial resolution and had smaller site- and county-level variations.

Our models demonstrate potential for predicting the occurrence of *Ae*. *aegypti* and *Ae*. *albopictus*, to better inform targeted mosquito control efforts. Model predictions produced with and without the benefit of recent surveillance data were of high accuracy suggesting that real-time forecasts could be produced with just climate data alone. Our results, however, call for the need for additional local data to explain a large spatial variation in mosquito occurrence and abundance.

## Supporting information

S1 Text Description of Mixed-effects zero-inflated negative binomial regression. (DOCX)

S1 Table Summary of other trap types included in the longitudinal training dataset. (DOCX)

S2 Table Odds ratio (OR) and incidence rate ratio (IRR) estimate from mixed-effects zero-inflated negative binomial analysis of covariates of Aedes trap rates in Florida using data from NOAA, from 2004 to 2018. (DOCX)

S3 Table Estimates of odds ratio (OR) and incidence rate ratio (IRR) from mixed-effects zero-inflated negative binomial analysis of *Aedes aegypti* and *Aedes albopictus* in Florida using data from Daymet, 2004–2018. (DOCX)

S4 Table Model performances on predicting occurrence and abundance varying from combinations of random effects and prior abundance information. (DOCX)

S5 Table Odds ratio (OR) and incidence rate ratio (IRR) estimate from mixed-effects zero-inflated negative binomial analysis of covariates of *Aedes aegypti* after removing data from Miami-Dade County. (DOCX)

S6 Table Model performances on predicting occurrence and abundance for spatial and temporal cross-validations. (DOCX)

S7 Table Model performances† on predicting occurrence and abundance for external testing dataset. (DOCX)

S8 Table Odds ratio (OR) and incidence rate ratio (IRR) estimate from mixed-effects zero-inflated negative binomial analysis of covariates of *Aedes aegypti* using mean temperature. (DOCX)

S9 Table Odds ratio (OR) and incidence rate ratio (IRR) estimate from mixed-effects zero-inflated negative binomial analysis of covariates of *Aedes aegypti* and *Aedes albopictus* collected from BG traps. (DOCX)

S10 Table Surveillance data by county. (DOCX)

S1 Fig Comparison of five datasets used in the study. (PNG)

S2 Fig Comparison of trap locations by longitudinal training dataset and external no abundance testing dataset. (PNG)

S3 Fig Spatial and temporal distribution of mosquito surveillance records from the longitudinal training dataset. (PNG)

S4 Fig Relations between occurrence and abundance of *Aedes aegypti* and *Aedes albopictus* with abiotic variables. (PNG)

S5 Fig Human population density (per km2) in Florida. (PNG)

S6 Fig Temporal variation in model predictions in abundance of *Aedes aegypti* (A) and *Aedes albopictus* (B).
(PNG)

S7 Fig Model performances on predicting occurrence (A and C) and abundance (B and D) for external testing dataset. Relative humidity minimum temperature and wind speed for each month of the year are shown in E, F and G.
(PNG)

S8 Fig Geographic distribution of mosquito trap types in the longitudinal training dataset. (PNG)

S9 Fig Correlation between predicted trap rate for *Aedes aegypti* using longitudinal data with and without data from Miami-Dade. Model incorporating both random effects and prior abundance information is used. (PNG)

S1 Video Weekly presence and absence of *Aedes aegypti* and *Aedes albopictus* in Florida. (MP4)

S2 Video Maps on predicted abundance of *Aedes aegypti* and *Aedes albopictus* in Florida, 2018 with high predictions in red and low predictions in blue. (MP4)

S3 Video Maps with predictions of abundance of *Aedes aegypti* (red) and *Aedes albopictus* (blue) for each month in 2018.
(MP4)
