# Supplementary material for: Modelling distributions of Aedes aegypti and Aedes albopictus using climate, host density and interspecies competition
Source: PLoS Negl Trop Dis. 2021 Mar 25;15(3):e0009063. doi: 10.1371/journal.pntd.0009063 (PMC8051819; doi:10.1371/journal.pntd.0009063)
Supplement: S1 Text — (DOCX) [file pntd.0009063.s001.docx]

## S1 Text

### Mixed-effects zero-inflated negative binomial regression. We applied the zero-inflated negative binomial regression (ZINB) to the surveillance data on a weekly basis. ZINB consists of two components, namely binary component (absence of mosquito) and negative binomial component (abundance of mosquito). We modelled the number ($\boldsymbol{Y}_{\boldsymbol{i,t,s}}$) of *Aedes aegypti* ($\boldsymbol{i=1}$) or *Aedes albopictus* ($\boldsymbol{i=2}$) collected by trap $\boldsymbol{s}$ at week $\boldsymbol{t}$ using the mixed-effects zero-inflated negative binomial (ZINB) regression. The expectation of $\boldsymbol{Y}_{\boldsymbol{i,t,s}}$ is modelled as follows:

$$E\left( Y_{i,t,s} \right) = \left( 1-\pi_{i,t,s} \right)E\left( Y_{i,t,s}|A=1 \right)+ \pi_{i,t,s}E\left( Y_{i,t,s}|A=0 \right)$$

where $A$ denotes the absence ($A=1$) and presence ($A=0$). $\pi_{i,t,s}$ denotes the probability of the presence of *Aedes* species, and follows the relation:

$$\pi_{i,t,s}= {\lambda_{i,t,s}}/\left( 1+\lambda_{i,t,s} \right)$$

$\lambda_{i,t,s}$ is derived from binary component of the ZINB model:

$$\log\left( \lambda_{i,t,s} \right)= \gamma_{0}+\sum_{m=t-3}^{t-1} \gamma_{i, m}I\left\{ Y_{i, m,s}>0 \right\}+ \sum_{m=t-3}^{t-1} \gamma_{j, m}I\left\{ Y_{j, m,s}>0 \right\}+\gamma_{D}D_{s}+\gamma_{T}T_{t,s}+ \gamma_{\Delta T}{\Delta T}_{t,s}+\gamma_{W}W_{t,s}+\gamma_{P}P_{t,s}+\gamma_{C}C_{t,s}+ \varepsilon_{z,s} + \varepsilon_{z,l}$$

where $I\{.\}$ denotes the indicator function. $D_{s}$ denotes the site-specific human population density, and the weekly site-specific meteorological factors are denoted by $W_{t,s}$ (wind speed), $T_{t,s}$ (minimum temperature), ${\Delta T}_{t,s}$ (maximum temperature) and $P_{t,s}$ (precipitation). $C_{t,s}$ denotes the trap type used in the trap episode. $\varepsilon$ represents the random effects across trap sites ($s$) and county ($l$) in the binary component ($z$) and negative binomial component ($n$):

$$\varepsilon\sim Normal\left( \alpha, \sigma^{2} \right)$$

$E\left( Y_{i,t,s}|A=1 \right)$ and E$\left( Y_{i,t,s}|A=0 \right)$ denotes the expectation of $Y_{i,t,s}$ when the *Aedes* mosquito is absent and present at the trap, respectively:

$$E\left( Y_{i,t,s}|A=1 \right)=0$$

and

$$E\left( Y_{i,t,s}|A=0 \right) \sim NB(d_{t, s}\mu_{i,t,s}, r)$$

$d_{t, s}$ denotes the trap-days of each trap episode and $r$ is the dispersal parameter of negative binomial distribution. $\mu_{i,t,s}$ is estimated from negative binomial component of ZINB regression:

$$\log(\mu_{i,t,s})=log\left( d_{t, s} \right)+\beta_{0}+ \sum_{m=t-3}^{t-1} \beta_{i,m}Y_{i,m,s}+ \sum_{m=t-3}^{t-1} \beta_{j,m}Y_{j,m,s} + \beta_{D}D_{s} + \beta_{T}T_{t,s} + \beta_{\Delta T}{\Delta T}_{t,s} + \beta_{W}W_{t,s} + \beta_{P}P_{t,s} + \beta_{c}C_{t,s} + \varepsilon_{n,s} + \varepsilon_{n,l}$$

## S1 Fig. Comparison of five datasets used in the study. (A) Full dataset is integrated from the statewide surveillance data. (B) Longitudinal training dataset is a subset of full dataset after excluding traps where four consecutive weeks of surveillance is available. (C) Spatial validation training dataset is a subset of the longitudinal by holding out a 10% randomly selected sites. Spatial validation used model trained by this dataset and tested by the traps shown in B but not in C. (D) Temporal validation training dataset is a subset of the longitudinal by holding out the data after 2017. Temporal validation used model trained by this dataset and tested by the traps shown in B but not in D. (E) No abundance testing dataset is a subset of full dataset, consisting of traps that are excluded by longitudinal training dataset. This dataset is used to test the predictions from “no abundance model” which is trained by the longitudinal training dataset. Maps produced using QGIS Version 3.0.2 (QGIS Development Team, 2018). Source of shapefile: Southwest Florida Water Management District (<https://geodata.myflorida.com/datasets/swfwmd::florida-counties>).

## S2 Fig. Comparison of trap locations by longitudinal training dataset and external no abundance testing dataset. Maps produced using QGIS Version 3.0.2 (QGIS Development Team, 2018). Source of shapefile: Southwest Florida Water Management District (https://geodata.myflorida.com/datasets/swfwmd::florida-counties).

## S3 Fig. Spatial and temporal distribution of mosquito surveillance records from the longitudinal training dataset. (A) *Aedes aegypti*. (B) *Aedes albopictus*. Trap sites and counties were ordered from north (upper) to south (lower). Heatmaps show weekly trap rate of each trap site. The sidebars indicate whether *Aedes aegypti* or *Aedes albopictus* had ever been reported by each site.

## S4 Fig. Relations between occurrence and abundance of *Aedes aegypti* and *Aedes albopictus* with abiotic variables. Values at x axis are the minimum, 25th quantile, median, 75th quantile and maximum value of the variable. Colored bar charts represent the proportion of occurrence reported by trap episodes. Colored box plots represent the median and interquartile range of the trap rate amongst traps where the vector occurred.

## S5 Fig. Human population density (per km^2^) in Florida. Maps produced using R Version 3.5.0 (R Foundation for Statistical Computing, Vienna, Austria). Source of raster: Socioeconomic Data and Applications Center (SEDAC) (http://sedac.ciesin.columbia.edu/data/collection/gpw-v4/sets/browse).

## S6 Fig. Temporal variation in model predictions in abundance of *Aedes aegypti* (A) and *Aedes albopictus* (B). Points are the median difference between predicted and observed abundance of *Aedes aegypti* and *Ae. albopictus* from the main analysis. Intervals are the 2.5% and 97.5% quantile of difference between predicted and observed abundance of the two *Aedes* species. Histograms are the monthly average of observed trap rates.

## S7 Fig. Model performances on predicting occurrence (A and C) and abundance (B and D) for external testing dataset. Model incorporating random effects but no prior abundance was fit to longitudinal training dataset. Predictions are calculated only using the fixed effects estimates derived from the model. External no abundance dataset contains those records in full dataset but were excluded from longitudinal training dataset. Strict external dataset is a subset of the external no abundance dataset and only contains records from counties that were not included in the longitudinal training dataset. Relative humidity minimum temperature and wind speed for each month of the year are shown in E, F and G.

## S8 Fig. Geographic distribution of mosquito trap types in the longitudinal training dataset. Maps produced using QGIS Version 3.0.2 (QGIS Development Team, 2018). Source of shapefile: Southwest Florida Water Management District (https://geodata.myflorida.com/datasets/swfwmd::florida-counties).

## S9 Fig. Correlation between predicted trap rate for *Aedes* *aegypti* using longitudinal data with and without data from Miami-Dade. Model incorporating both random effects and prior abundance information is used.

## S1 Video. Weekly presence and absence of *Aedes aegypti* and *Aedes albopictus* in Florida. Maps produced using R Version 3.5.0 (R Foundation for Statistical Computing, Vienna, Austria). Source of shapefile: Southwest Florida Water Management District (https://geodata.myflorida.com/datasets/swfwmd::florida-counties.

**S2 Video. Maps on predicted abundance of *Aedes aegypti* (red) and *Aedes albopictus* (blue) in Florida, 2018.** Predictions are derived from “no abundance model”. Parts A and B show results incorporating random effects representing differences in trapping counts by county. Parts C and D show results only incorporating fixed effects. Maps produced using R Version 3.5.0 (R Foundation for Statistical Computing, Vienna, Austria). Source of shapefile: Southwest Florida Water Management District (https://geodata.myflorida.com/datasets/swfwmd::florida-counties).
