# Supplementary material for: Modelling distributions of Aedes aegypti and Aedes albopictus using climate, host density and interspecies competition
Source: PLoS Negl Trop Dis. 2021 Mar 25;15(3):e0009063. doi: 10.1371/journal.pntd.0009063 (PMC8051819; doi:10.1371/journal.pntd.0009063)
Supplement: S1 Table — (DOCX) [file pntd.0009063.s002.docx]

## S1 Table. Summary of other trap types included in the longitudinal training dataset.

| **Trap types** | **Number of records** |
| --- | --- |
| **BG sentinel trap** | 9,518 (7.2%) |
| **Light trap** | 107,571 (81.4%) |
| CDC light traps | 95,554 (88.8%) |
| New Jersey light traps | 8,451 (7.9%) |
| Non-specific light traps | 3,566 (3.3%) |
| **Other trap types** | 14,999 (11.4%) |
| Mosquito magnet | 3,545 (23.6%) |
| Suction trap | 3,372 (22.5%) |
| Propane | 2,676 (17.8%) |
| ABC | 1,920 (12.8%) |
| Gravid trap | 1,827 (12.2%) |
| Exit | 1,178 (7.9%) |
| Route | 268 (1.8%) |
| Unknown | 139 (0.9%) |
| Fay prince | 64 (0.4%) |
| Wilton trap | 10 (0.1%) |
