# Supplementary material for: Modelling distributions of Aedes aegypti and Aedes albopictus using climate, host density and interspecies competition
Source: PLoS Negl Trop Dis. 2021 Mar 25;15(3):e0009063. doi: 10.1371/journal.pntd.0009063 (PMC8051819; doi:10.1371/journal.pntd.0009063)
Supplement: S2 Table — (DOCX) [file pntd.0009063.s003.docx]

## S2 Table. Odds ratio (OR) and incidence rate ratio (IRR) estimate from mixed-effects zero-inflated negative binomial analysis of covariates of *Aedes* trap rates in Florida using data from NOAA, from 2004 to 2018.

| **Variables** | ***Aedes aegypti*** | | ***Aedes albopictus*** | |
| --- | --- | --- | --- | --- |
|  | **OR**  **(95% CI^†^)** | **IRR**  **(95% CI^†^)** | **OR**  **(95% CI^†^)** | **IRR**  **(95% CI^†^)** |
| **Previous *Ae. aegypti* abundance/presence**  **(per trap-day)** |  |  |  |  |
| Trap rate in week *t-1* | 2.44  (2.18, 2.74)* | 1.03  (1.02, 1.03)* | 1.22  (1.11, 1.35)* | 1.00  (1.00, 1.01) |
| Trap rate in week *t-2* | 2.42  (2.16, 2.71)* | 1.03  (1.03, 1.03)* | 1.43  (1.29, 1.58)* | 1.00  (0.99, 1.00) |
| Trap rate in week *t-3* | 1.81  (1.61, 2.03)* | 1.02  (1.01, 1.02)* | 1.05  (0.95, 1.16) | 1.00  (1.00, 1.01) |
| **Previous *Ae. albopictus* abundance/presence**  **(per trap-day)** |  |  |  |  |
| Trap rate in week *t-1* | 1.30  (1.16, 1.46)* | 0.99  (0.99, 1.00)* | 2.51  (2.35, 2.68)* | 1.02  (1.02, 1.03)* |
| Trap rate in week *t-2* | 1.46  (1.30, 1.64)* | 0.99  (0.99, 1.00)* | 2.23  (2.08, 2.38)* | 1.02  (1.01, 1.02)* |
| Trap rate in week *t-3* | 1.28  (1.14, 1.43)* | 0.99  (0.99, 1.00)* | 1.70  (1.59, 1.82)* | 1.02  (1.01, 1.02)* |
| **Human population density (**$\boldsymbol{100 per k}\boldsymbol{m}^{\boldsymbol{2}}$**)** | 1.05  (1.03, 1.07)* | 1.01  (0.99, 1.02) | 0.95  (0.94, 0.97)* | 0.98  (0.97, 1.00)* |
| **Meteorology** |  |  |  |  |
| Average wind speed  ($m/s$) | 0.98  (0.95, 1.00)* | 0.97  (0.96, 0.98)* | 0.97  (0.96, 0.98)* | 0.96  (0.95, 0.97)* |
| Minimum temperature  ($℃$) | 1.01  (1.00, 1.02) | 1.11  (1.10, 1.11)* | 1.07  (1.06, 1.07)* | 1.08  (1.07, 1.08)* |
| Residuals of maximum  temperature  ($℃$) | 1.00  (0.96, 1.03) | 1.08  (1.06, 1.11)* | 1.09  (1.06, 1.11)* | 1.06  (1.04, 1.08)* |
| Precipitation  ($mm$) | 0.85  (0.69, 1.05) | 1.42  (1.26, 1.59)* | 1.05  (0.94, 1.19) | 1.09  (0.99, 1.19) |
| **Trap type** |  |  |  |  |
| BG sentinel | Ref. | Ref. | Ref. | Ref. |
| Light trap | 0.00  (0.00, 0.01)* | 0.40  (0.31, 0.52)* | 0.78  (0.60, 1.01) | 0.30  (0.24, 0.37)* |
| Other | 0.01  (0.00, 0.02)* | 0.20  (0.14, 0.29)* | 1.76  (1.28, 2.43)* | 0.26  (0.20, 0.33)* |
| **Random effects** |  |  |  |  |
| Site | 1.32 | 1.66 | 1.38 | 0.90 |
| County | 12.24 | 2.82 | 6.57 | 1.63 |
| **Dispersion parameter** | -- | 1.45  (1.41, 1.50) | -- | 1.13  (1.10, 1.17) |

* P < 0.05. **^†^** Credible interval
