# Supplementary material for: Modelling distributions of Aedes aegypti and Aedes albopictus using climate, host density and interspecies competition
Source: PLoS Negl Trop Dis. 2021 Mar 25;15(3):e0009063. doi: 10.1371/journal.pntd.0009063 (PMC8051819; doi:10.1371/journal.pntd.0009063)
Supplement: S3 Table — (DOCX) [file pntd.0009063.s004.docx]

## S3 Table . Estimates of odds ratio (OR) and incidence rate ratio (IRR) from mixed-effects zero-inflated negative binomial analysis of *Aedes* *aegypti* and *Aedes albopictus* in Florida using data from Daymet, 2004-2018.

| **Variables** | ***Aedes aegypti*** | | ***Aedes albopictus*** | |
| --- | --- | --- | --- | --- |
|  | **OR**  **(95% CI^†^)** | **IRR**  **(95% CI^†^)** | **OR**  **(95% CI^†^)** | **IRR**  **(95% CI^†^)** |
| **Previous *Ae. aegypti* abundance/presence**  **(per trap-day)** |  |  |  |  |
| Trap rate in week *t-1* | 3.168 (1.923, 5.212)*** | 1.060 (1.040, 1.079)*** | 1.468 (1.026, 2.102)* | 0.993 (0.976, 1.010) |
| Trap rate in week *t-2* | 3.414 (2.088, 5.579)*** | 1.057 (1.039, 1.075)*** | 1.793 (1.260, 2.552)** | 0.995 (0.978, 1.012) |
| Trap rate in week *t-3* | 3.834 (2.199, 6.686)*** | 1.012 (0.994, 1.030) | 1.084 (0.772, 1.523) | 1.007 (0.990, 1.025) |
| **Previous *Ae. albopictus* abundance/presence**  **(per trap-day)** |  |  |  |  |
| Trap rate in week *t-1* | 1.266 (0.774, 2.069) | 0.996 (0.971, 1.020) | 4.380 (3.401, 5.635)*** | 1.030 (1.016, 1.045)*** |
| Trap rate in week *t-2* | 1.517 (0.946, 2.435) | 1.005 (0.982, 1.028) | 3.494 (2.721, 4.486)*** | 1.023 (1.009, 1.037)** |
| Trap rate in week *t-3* | 1.069 (0.660, 1.733) | 1.003 (0.979, 1.027) | 2.404 (1.876, 3.077)*** | 1.029 (1.015, 1.044)*** |
| **Human population density**  **(**$\boldsymbol{100 persons per k}\boldsymbol{m}^{\boldsymbol{2}}$**)** | 1.060 (1.020, 1.102)** | 0.992 (0.977, 1.006) | 0.972 (0.952, 0.994)* | 0.992 (0.973, 1.012) |
| **Meteorology** |  |  |  |  |
| Average wind speed ($m/s$) | 0.945 (0.833, 1.071) | 0.894 (0.834, 0.958)** | 0.903 (0.834, 0.978)* | 0.870 (0.815, 0.928)*** |
| Minimum temperature ($℃$) | 0.952 (0.914, 0.992)* | 1.076 (1.052, 1.100)*** | 1.024 (0.998, 1.051) | 1.064 (1.041, 1.087)*** |
| Residual of maximum temperature ($℃$) | 0.996 (0.907, 1.093) | 1.022 (0.969, 1.079) | 0.996 (0.941, 1.054) | 1.038 (0.993, 1.085) |
| Precipitation | 1.003 (0.991, 1.014) | 1.001 (0.996, 1.006) | 0.998 (0.991, 1.005) | 1.002 (0.998, 1.006) |
| **Trap type** |  |  |  |  |
| BG sentinel | Ref. | Ref. | Ref. | Ref. |
| Light trap | 0.060 (0.011, 0.331)** | 0.373 (0.247, 0.564)*** | 2.090 (1.310, 3.330)** | 0.335 (0.219, 0.511)*** |
| Other | 0.165 (0.023, 1.203) | 0.128 (0.052, 0.316)*** | 3.187 (1.499, 6.780)** | 0.211 (0.112, 0.396)*** |
| **Random effects** |  |  |  |  |
| Site | 0.56 | 0.67 | 0.34 | 0.42 |
| County | 2.02 | 1.86 | 1.30 | 1.39 |
| **Dispersion parameter** | -- | 0.703 (0.403, 1.227) | -- | 0.876 (0.577, 1.330) |
