# Supplementary material for: Modelling distributions of Aedes aegypti and Aedes albopictus using climate, host density and interspecies competition
Source: PLoS Negl Trop Dis. 2021 Mar 25;15(3):e0009063. doi: 10.1371/journal.pntd.0009063 (PMC8051819; doi:10.1371/journal.pntd.0009063)
Supplement: S4 Table — (DOCX) [file pntd.0009063.s005.docx]

## S4 Table. Model performances on predicting occurrence and abundance varying from combinations of random effects and prior abundance information.

|  | ***Aedes aegypti*** | ***Aedes albopictus*** |
| --- | --- | --- |
| **Consistent with observed presence** | | |
| Random effect + prior abundance | 91.1 (91.0, 91.3) | 84.9 (84.7, 85.1) |
| Random effect only | 90.3 (90.1, 90.5) | 82.9 (82.7, 83.1) |
| Prior abundance only | 83.4 (83.2, 83.6) | 72.1 (71.9, 72.3) |
| None | 76.1 (75.9, 76.4) | 58.8 (58.5, 59.0) |
| **Consistent with observed abundance where present** | | |
| Random effect + prior abundance | 78.7 (77.9, 79.4) | 84.9 (84.3, 85.5) |
| Random effect only | 78.3 (77.5, 79.1) | 84.1 (83.5, 84.8) |
| Prior abundance only | 73.3 (72.5, 74.1) | 80.3 (79.7, 81.0) |
| None | 75.2 (74.4, 75.9) | 89.2 (88.5, 89.7) |

Proportions are shown in the table.
