# Supplementary material for: Modelling distributions of Aedes aegypti and Aedes albopictus using climate, host density and interspecies competition
Source: PLoS Negl Trop Dis. 2021 Mar 25;15(3):e0009063. doi: 10.1371/journal.pntd.0009063 (PMC8051819; doi:10.1371/journal.pntd.0009063)
Supplement: S5 Table — (DOCX) [file pntd.0009063.s006.docx]

## S5 Table. Odds ratio (OR) and incidence rate ratio (IRR) estimate from mixed-effects zero-inflated negative binomial analysis of covariates of *Aedes aegypti* after removing data from Miami-Dade County.

| **Variables** | ***Aedes aegypti*** | |
| --- | --- | --- |
|  | **OR (95% CI^†^)** | **IRR (95% CI^†^)** |
| **Previous *Ae. Aegypti* abundance**  **(per trap-day)** |  |  |
| Trap rate of *Ae. aegypti* in week t-1 | 2.79  (2.41, 3.23)* | 1.03  (1.03, 1.04)* |
| Trap rate of *Ae. aegypti* in week t-2 | 2.26  (1.95, 2.61)* | 1.03  (1.03, 1.04)* |
| Trap rate of *Ae. aegypti* in week t-3 | 2.12  (1.83, 2.46)* | 1.02  (1.01, 1.03)* |
| **Previous *Ae. Albopictus* abundance**  **(per trap-day)** |  |  |
| Trap rate of *Ae. albopictus* in week t-1 | 1.45  (1.27, 1.66)* | 1.00  (0.99, 1.00) |
| Trap rate of *Ae. albopictus* in week t-2 | 1.48  (1.29, 1.70)* | 0.99  (0.98, 1.00)*^†^ |
| Trap rate of *Ae. albopictus* in week t-3 | 1.41  (1.23, 1.62)* | 1.00  (0.99, 1.00) |
| **Human population density (100 per** $\boldsymbol{k}\boldsymbol{m}^{\boldsymbol{2}}$**)** | 1.12  (1.09, 1.16)* | 1.08  (1.04, 1.12)* |
| **Meteorology** |  |  |
| Average wind speed ($m/s$) | 1.03  (0.99, 1.08) | 0.90  (0.88, 0.93)* |
| Minimum temperature ($℃$) | 1.03  (1.01, 1.04)* | 1.09  (1.08, 1.10)* |
| Residuals of maximum temperature ($℃$) | 1.02  (0.92, 1.13) | 1.03  (0.96, 1.10) |
| Relative humidity ($mm$) | 1.00  (0.98, 1.01) | 1.01  (1.00, 1.02)*^†^ |
| **Random effects** |  |  |
| Site | 1.03 | 2.28 |
| County | 10.90 | 3.39 |
| **Dispersion parameter** | -- | 1.89 (1.78, 2.00) |

* P < 0.05. **^†^** Credible interval. † The values with three effective digits for these estimations are: 0.991 (0.984, 0.998) and 1.013 (1.003, 1.023).
