# Supplementary material for: Modelling distributions of Aedes aegypti and Aedes albopictus using climate, host density and interspecies competition
Source: PLoS Negl Trop Dis. 2021 Mar 25;15(3):e0009063. doi: 10.1371/journal.pntd.0009063 (PMC8051819; doi:10.1371/journal.pntd.0009063)
Supplement: S6 Table — (DOCX) [file pntd.0009063.s007.docx]

## S6 Table. Model performances on predicting occurrence and abundance for spatial and temporal cross-validations.

|  | ***Aedes aegypti*** | ***Aedes albopictus*** |
| --- | --- | --- |
| **Consistent with observed presence** | | |
| Spatial validation | 93.1 (92.6, 93.5) | 83.9 (83.3, 84.5) |
| Temporal validation | 84.5 (83.1, 85.9) | 95.3 (94.4, 96.1) |
| **Consistent with observed abundance where present** | | |
| Spatial validation | 72.1 (69.3, 74.9) | 75.3 (72.9, 77.6) |
| Temporal validation | 91.1 (87.9, 93.7) | 100.0 (86.8, 100.0) |

Proportions are shown in the table.­
