# Supplementary material for: Modelling distributions of Aedes aegypti and Aedes albopictus using climate, host density and interspecies competition
Source: PLoS Negl Trop Dis. 2021 Mar 25;15(3):e0009063. doi: 10.1371/journal.pntd.0009063 (PMC8051819; doi:10.1371/journal.pntd.0009063)
Supplement: S7 Table — (DOCX) [file pntd.0009063.s008.docx]

## S7 Table. Model performances^†^ on predicting occurrence and abundance for external testing dataset.

|  | ***Aedes aegypti*** | ***Aedes albopictus*** |
| --- | --- | --- |
| **Consistent with observed presence** | | |
| External^§^ | 88.8 (88.8, 88.9) | 83.2 (83.1, 83.3) |
| Strict external^#^ | 84.2 (83.2, 85.2) | 61.9 (60.2, 64.2) |
| **Consistent with observed abundance where present** | | |
| External^§^ | 61.0 (60.4, 61.7) | 80.8 (79.7, 81.9) |
| Strict external^#^ | 48.3 (33.9, 63.0) | 60.0 (41.6, 78.8) |

**†** Model incorporating random effects but no prior abundance was fit to longitudinal training dataset. Predictions are calculated only using the fixed effects estimates derived from the model.

§ External no abundance dataset contains those records in full dataset but were excluded from longitudinal training dataset.

# Strict external dataset is a subset of the external no abundance dataset and only contains records from counties that were not included in the longitudinal training dataset.
