# Supplementary material for: Modelling distributions of Aedes aegypti and Aedes albopictus using climate, host density and interspecies competition
Source: PLoS Negl Trop Dis. 2021 Mar 25;15(3):e0009063. doi: 10.1371/journal.pntd.0009063 (PMC8051819; doi:10.1371/journal.pntd.0009063)
Supplement: S8 Table — (DOCX) [file pntd.0009063.s009.docx]

## S8 Table. Odds ratio (OR) and incidence rate ratio (IRR) estimate from mixed-effects zero-inflated negative binomial analysis of covariates of *Aedes aegypti* using mean temperature.

| **Variables** | ***Aedes aegypti*** | | ***Aedes albopictus*** | |
| --- | --- | --- | --- | --- |
|  | **OR**  **(95% CI^†^)** | **IRR**  **(95% CI^†^)** | **OR**  **(95% CI^†^)** | **IRR**  **(95% CI^†^)** |
| **Previous *Ae. aegypti* abundance/presence**  **(per trap-day)** |  |  |  |  |
| Trap rate in week *t-1* | 2.44  (2.18, 2.74)* | 1.03  (1.03, 1.03)* | 1.21  (1.10, 1.34)* | 1.00  (1.00, 1.01) |
| Trap rate in week *t-2* | 2.38  (2.13, 2.67)* | 1.03  (1.03, 1.03)* | 1.42  (1.28, 1.57)* | 1.00  (0.99, 1.00) |
| Trap rate in week *t-3* | 1.84  (1.64, 2.07)* | 1.02  (1.01, 1.02)* | 1.04  (0.94, 1.15) | 1.00  (1.00, 1.01) |
| **Previous *Ae. albopictus* abundance/presence**  **(per trap-day)** |  |  |  |  |
| Trap rate in week *t-1* | 1.30  (1.16, 1.47)* | 0.99  (0.99, 1.00)* | 2.48  (2.32, 2.65)* | 1.02  (1.02, 1.03)* |
| Trap rate in week *t-2* | 1.44  (1.29, 1.62)* | 0.99  (0.99, 1.00)* | 2.19  (2.05, 2.35)* | 1.02  (1.01, 1.02)* |
| Trap rate in week *t-3* | 1.28  (1.14, 1.44)* | 0.99  (0.99, 1.00)* | 1.68  (1.57, 1.80)* | 1.02  (1.01, 1.02)* |
| **Human population density (**$\boldsymbol{100 per k}\boldsymbol{m}^{\boldsymbol{2}}$**)** | 1.05  (1.03, 1.07)* | 1.01  (0.99, 1.02) | 0.95  (0.94, 0.97)* | 0.98  (0.97, 1.00)* |
| **Meteorology** |  |  |  |  |
| Average wind speed ($m/s$) | 0.98  (0.95, 1.01) | 0.97  (0.95, 0.98)* | 0.97  (0.95, 0.99)* | 0.97  (0.95, 0.98)* |
| Mean temperature ($℃$) | 1.01  (1.00, 1.02) | 1.13  (1.13, 1.14)* | 1.09  (1.08, 1.10)* | 1.10  (1.09, 1.11)* |
| Precipitation ($mm$) | 1.00  (0.99, 1.01) | 1.01  (1.00, 1.01)* | 0.99  (0.99, 1.00)* | 1.00  (0.99, 1.00) |
| **Trap type** |  |  |  |  |
| BG sentinel | Ref. | Ref. | Ref. | Ref. |
| Light trap | 0.00  (0.00, 0.01)* | 0.40  (0.31, 0.52)* | 0.78  (0.60, 1.00) | 0.29  (0.24, 0.36)* |
| Other | 0.01  (0.00, 0.02)* | 0.19  (0.13, 0.28)* | 1.77  (1.28, 2.44)* | 0.25  (0.19, 0.33)* |
| **Random effects** |  |  |  |  |
| Site | 1.34 | 1.66 | 1.40 | 0.90 |
| County | 12.32 | 2.79 | 6.60 | 1.56 |
| **Dispersion parameter** | -- | 1.47  (1.43, 1.52) | -- | 1.13  (1.10, 1.17) |
