# Supplementary material for: Modelling distributions of Aedes aegypti and Aedes albopictus using climate, host density and interspecies competition
Source: PLoS Negl Trop Dis. 2021 Mar 25;15(3):e0009063. doi: 10.1371/journal.pntd.0009063 (PMC8051819; doi:10.1371/journal.pntd.0009063)
Supplement: S9 Table — (DOCX) [file pntd.0009063.s010.docx]

## S9 Table. Odds ratio (OR) and incidence rate ratio (IRR) estimate from mixed-effects zero-inflated negative binomial analysis of covariates of *Aedes aegypti* and *Aedes albopictus* collected from BG traps.

| **Variables** | ***Aedes aegypti*** | | ***Aedes albopictus*** | |
| --- | --- | --- | --- | --- |
|  | **OR**  **(95% CI^†^)** | **IRR**  **(95% CI^†^)** | **OR**  **(95% CI^†^)** | **IRR**  **(95% CI^†^)** |
| **Previous *Ae. aegypti* abundance/presence**  **(per trap-day)** |  |  |  |  |
| Trap rate in week *t-1* | 5.16  (2.45, 10.87)* | 1.04  (1.03, 1.04)* | 0.99  (0.59, 1.67) | 1.00  (0.99, 1.02) |
| Trap rate in week *t-2* | 6.61  (3.48, 12.54)* | 1.02  (1.02, 1.03)* | 1.10  (0.64, 1.89) | 1.01  (0.99, 1.03) |
| Trap rate in week *t-3* | 8.58  (4.13, 17.85)* | 1.02  (1.01, 1.02)* | 0.65  (0.40, 1.08) | 1.00  (0.98, 1.02) |
| **Previous *Ae. albopictus* abundance/presence**  **(per trap-day)** |  |  |  |  |
| Trap rate in week *t-1* | 0.42  (0.22, 0.80)* | 1.02  (1.00, 1.03)* | 3.27  (1.98, 5.41)* | 1.06  (1.04, 1.07)* |
| Trap rate in week *t-2* | 0.96  (0.49, 1.90) | 0.99  (0.98, 1.01) | 1.12  (0.70, 1.79) | 1.06  (1.04, 1.07)* |
| Trap rate in week *t-3* | 0.78  (0.41, 1.50) | 0.99  (0.98, 1.01) | 3.00  (1.89, 4.77)* | 1.04  (1.02, 1.05)* |
| **Human population density (**$\boldsymbol{100 per k}\boldsymbol{m}^{\boldsymbol{2}}$**)** | 1.12  (1.03, 1.23)* | 0.99  (0.98, 1.01) | 0.94  (0.90, 0.97)* | 0.99  (0.96, 1.02) |
| **Meteorology** |  |  |  |  |
| Average wind speed  ($m/s$) | 1.16  (0.99, 1.35) | 0.97  (0.96, 0.99)* | 1.02  (0.92, 1.12) | 0.91  (0.87, 0.96)* |
| Minimum temperature ($℃$) | 1.02  (0.93, 1.13) | 1.12  (1.11, 1.13)* | 1.02  (0.97, 1.07) | 1.10  (1.07, 1.12)* |
| Residuals of maximum temperature ($℃$) | 1.25  (0.92, 1.70) | 1.07  (1.04, 1.10)* | 1.02  (0.86, 1.21) | 1.07  (0.99, 1.16) |
| Relative humidity ($\%$) | 0.31  (0.06, 1.53) | 1.58  (1.32, 1.89)* | 0.39  (0.18, 0.83)* | 1.27  (0.93, 1.73) |
| **Random effects** |  |  |  |  |
| Site | 0.30 | 0.60 | 0.87 | 0.57 |
| County | 2.36 | 0.81 | 4.30 | 1.19 |
| **Dispersion parameter** | -- | 1.35  (1.31, 1.40) | -- | 1.10  (0.98, 1.23) |

* P < 0.05. **^†^** Credible interval
