# Supplementary material for: Modelling distributions of Aedes aegypti and Aedes albopictus using climate, host density and interspecies competition
Source: PLoS Negl Trop Dis. 2021 Mar 25;15(3):e0009063. doi: 10.1371/journal.pntd.0009063 (PMC8051819; doi:10.1371/journal.pntd.0009063)
Supplement: S10 Table — (DOCX) [file pntd.0009063.s011.docx]

## S10 Table. Surveillance data by county.

| **County** | **Full** | **Longitudinal Training** | **Spatial Training** | **Temporal Training** | **No Abundance Testing** |
| --- | --- | --- | --- | --- | --- |
| Hillsborough | 24854  (14.0%) | 16475 (12.5%) | 14861 (12.5%) | 16475 (12.7%) | 8379  (18.4%) |
| Pinellas | 22335 (12.6%) | 20058 (15.2%) | 18607 (15.7%) | 19673 (15.2%) | 2277  (5.0%) |
| St. Johns | 21872 (12.3%) | 17751 (13.4%) | 15954 (13.4%) | 17751 (13.7%) | 4121  (9.1%) |
| Polk | 20751 (11.7%) | 15543 (11.8%) | 12528 (10.5%) | 15543 (12.0%) | 5208  (11.4%) |
| Dade | 18634 (10.5%) | 14980 (11.3%) | 14129 (11.9%) | 14158 (10.9%) | 3654  (8.0%) |
| Lee | 13812 (7.8%) | 8045 (6.1%) | 6613 (5.6%) | 8045 (6.2%) | 5767 (12.7%) |
| Citrus | 8471 (4.8%) | 6959 (5.3%) | 6695 (5.6%) | 6959 (5.4%) | 1512 (3.3%) |
| Walton | 8380 (4.7%) | 6186 (4.7%) | 5890 (5.0%) | 6106 (4.7%) | 2194 (4.8%) |
| Palm Beach | 7864 (4.4%) | 7008 (5.3%) | 6551 (5.5%) | 6912 (5.3%) | 856 (1.9%) |
| Pasco | 5722 (3.2%) | 3468 (2.6%) | 2922 (2.5%) | 3468 (2.7%) | 2254 (5.0%) |
| Osceola | 4522 (2.5%) | 2203 (1.7%) | 1989 (1.7%) | 2203 (1.7%) | 2319 (5.1%) |
| St. Lucie | 3717 (2.1%) | 2746 (2.1%) | 2527 (2.1%) | 2493 (1.9%) | 971 (2.1%) |
| Flagler | 3715 (2.1%) | 3150 (2.4%) | 2880 (2.4%) | 3118 (2.4%) | 565 (1.2%) |
| Martin | 2660 (1.5%) | 2561 (1.9%) | 2453 (2.1%) | 2350 (1.8%) | 99 (0.2%) |
| Alachua | 2015 (1.1%) | 1538 (1.2%) | 1194 (1.0%) | 1538 (1.2%) | 477 (1.0%) |
| Hernando | 1868 (1.1%) | 1526 (1.2%) | 1299 (1.1%) | 1526 (1.2%) | 342 (0.8%) |
| Hendry | 1052 (0.6%) | 437 (0.3%) | 337 (0.3%) | 437 (0.3%) | 615 (1.4%) |
| Sarasota | 1004 (0.6%) | 268 (0.2%) | 249 (0.2%) | 268 (0.2%) | 736 (1.6%) |
| Bay | 933 (0.5%) | 137 (0.1%) | 105 (0.1%) | 137 (0.1%) | 796 (1.7%) |
| Orange | 724 (0.4%) | 35 (0%) | 35 (0%) | 35 (0%) | 689 (1.5%) |
| Okaloosa | 324 (0.2%) | 216 (0.2%) | 216 (0.2%) | 132 (0.1%) | 108 (0.2%) |
| Santa Rosa | 324 (0.2%) | 168 (0.1%) | 168 (0.1%) | 108 (0.1%) | 156 (0.3%) |
| Brevard | 176 (0.1%) | 30 (0%) | 25 (0%) | 30 (0%) | 146 (0.3%) |
| Holmes | 156 (0.1%) | 84 (0.1%) | 84 (0.1%) | 0 (0%) | 72 (0.2%) |
| Liberty | 150 (0.1%) | 84 (0.1%) | 77 (0.1%) | 0 (0%) | 66 (0.1%) |
| Madison | 149 (0.1%) | 44 (0%) | 40 (0%) | 0 (0%) | 105 (0.2%) |
| Bradford | 137 (0.1%) | 0 (0%) | 0 (0%) | 0 (0%) | 137 (0.3%) |
| Wakulla | 132 (0.1%) | 82 (0.1%) | 82 (0.1%) | 0 (0%) | 50 (0.1%) |
| Indian River | 130 (0.1%) | 0 (0%) | 0 (0%) | 0 (0%) | 130 (0.3%) |
| Washington | 130 (0.1%) | 38 (0%) | 26 (0%) | 0 (0%) | 92 (0.2%) |
| Taylor | 120 (0.1%) | 84 (0.1%) | 84 (0.1%) | 0 (0%) | 36 (0.1%) |
| Gadsden | 108 (0.1%) | 48 (0%) | 44 (0%) | 0 (0%) | 60 (0.1%) |
| Jackson | 108 (0.1%) | 56 (0%) | 53 (0%) | 0 (0%) | 52 (0.1%) |
| Jefferson | 108 (0.1%) | 41 (0%) | 33 (0%) | 0 (0%) | 67 (0.1%) |
| Calhoun | 99 (0.1%) | 39 (0%) | 31 (0%) | 0 (0%) | 60 (0.1%) |
| Collier | 91 (0.1%) | 0 (0%) | 0 (0%) | 0 (0%) | 91 (0.2%) |
| Gulf | 65 (0%) | 0 (0%) | 0 (0%) | 0 (0%) | 65 (0.1%) |
| Charlotte | 57 (0%) | 0 (0%) | 0 (0%) | 0 (0%) | 57 (0.1%) |
| Escambia | 40 (0%) | 0 (0%) | 0 (0%) | 0 (0%) | 40 (0.1%) |
| Okeechobee | 31 (0%) | 0 (0%) | 0 (0%) | 0 (0%) | 31 (0.1%) |
| Union | 15 (0%) | 0 (0%) | 0 (0%) | 0 (0%) | 15 (0%) |
| Leon | 13 (0%) | 0 (0%) | 0 (0%) | 0 (0%) | 13 (0%) |
| Baker | 10 (0%) | 0 (0%) | 0 (0%) | 0 (0%) | 10 (0%) |
| Dixie | 10 (0%) | 0 (0%) | 0 (0%) | 0 (0%) | 10 (0%) |
| Gilchrist | 10 (0%) | 0 (0%) | 0 (0%) | 0 (0%) | 10 (0%) |
| Marion | 10 (0%) | 0 (0%) | 0 (0%) | 0 (0%) | 10 (0%) |
| Suwannee | 10 (0%) | 0 (0%) | 0 (0%) | 0 (0%) | 10 (0%) |
| Nassau | 5 (0%) | 0 (0%) | 0 (0%) | 0 (0%) | 5 (0%) |
| Total | 177623 | 132088 | 118781 | 129465 | 45535 |
