# Supplementary figures and images for: Modelling distributions of Aedes aegypti and Aedes albopictus using climate, host density and interspecies competition
Source: PLoS Negl Trop Dis. 2021 Mar 25;15(3):e0009063. doi: 10.1371/journal.pntd.0009063 (PMC8051819; doi:10.1371/journal.pntd.0009063)

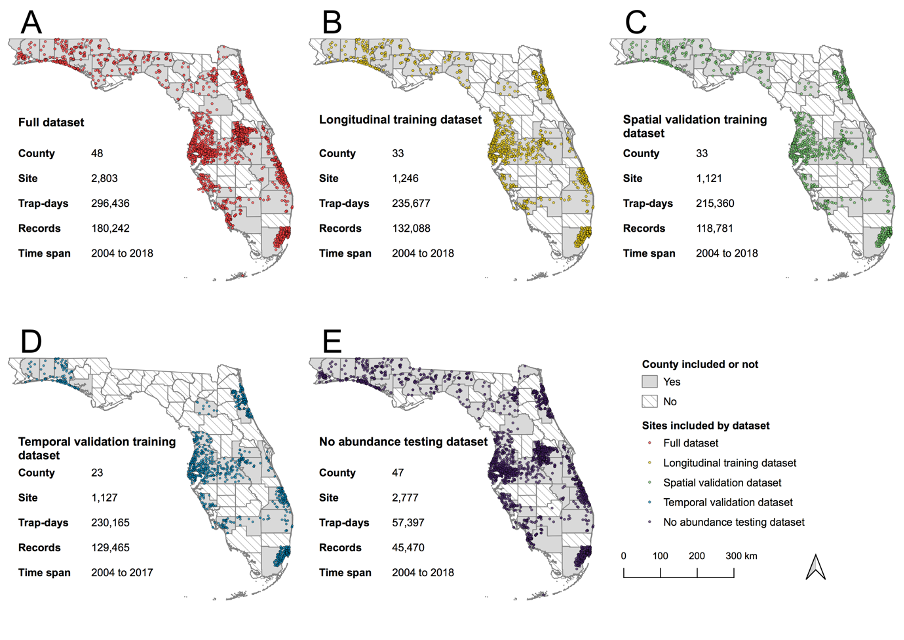

Supplement: S1 Fig — (PNG) [file pntd.0009063.s012.png]

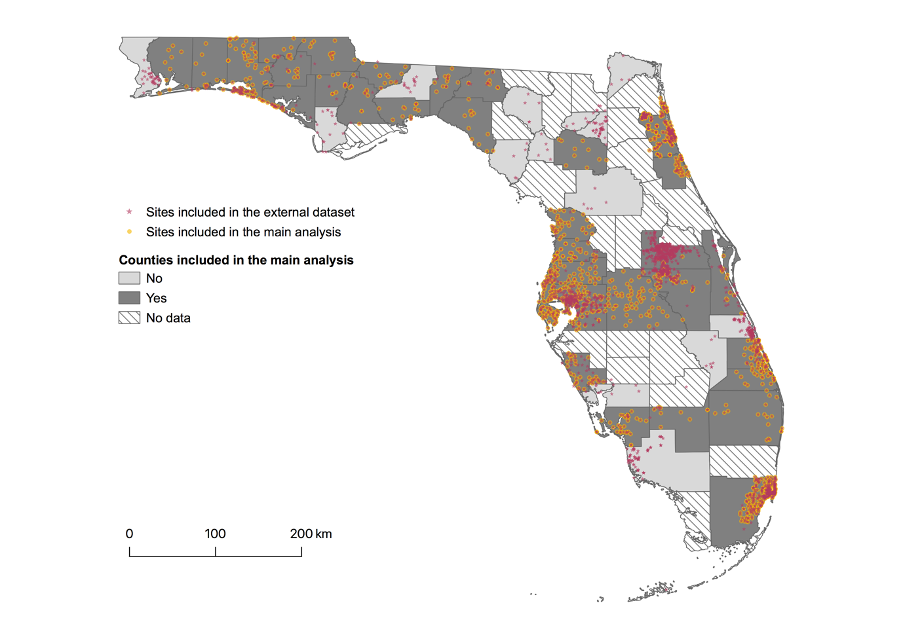

Supplement: S2 Fig — (PNG) [file pntd.0009063.s013.png]

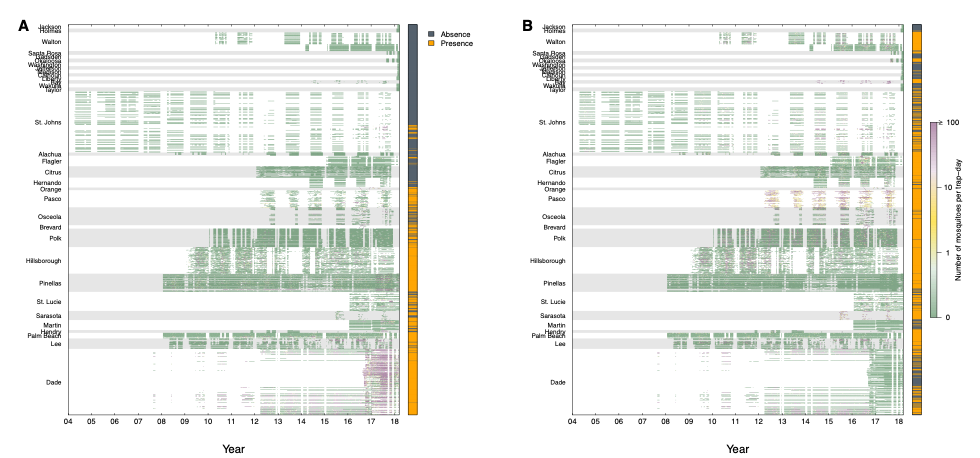

Supplement: S3 Fig — (PNG) [file pntd.0009063.s014.png]

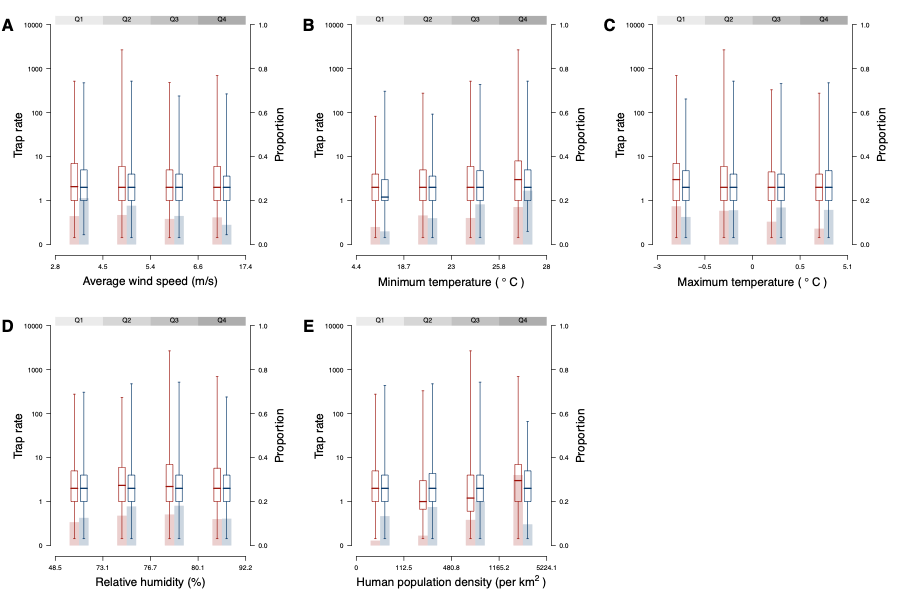

Supplement: S4 Fig — (PNG) [file pntd.0009063.s015.png]

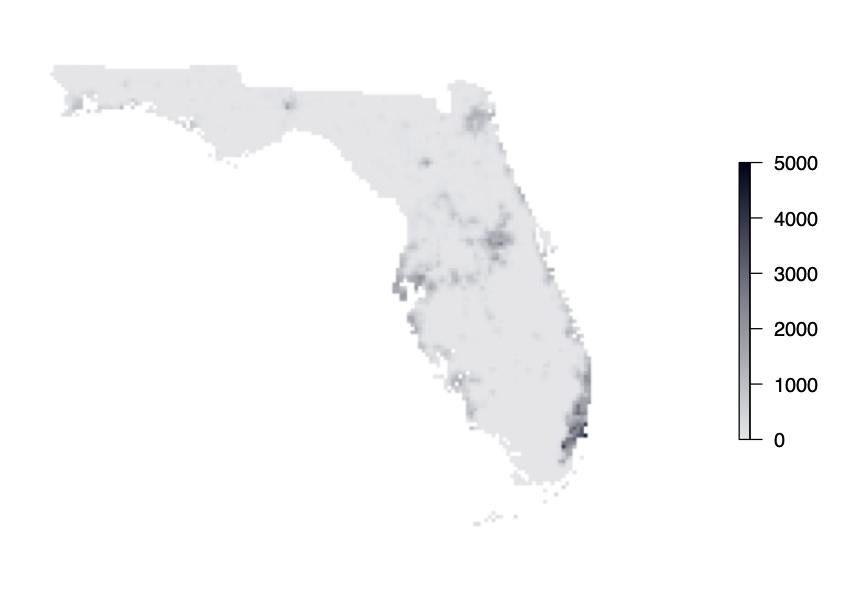

Supplement: S5 Fig — (PNG) [file pntd.0009063.s016.png]

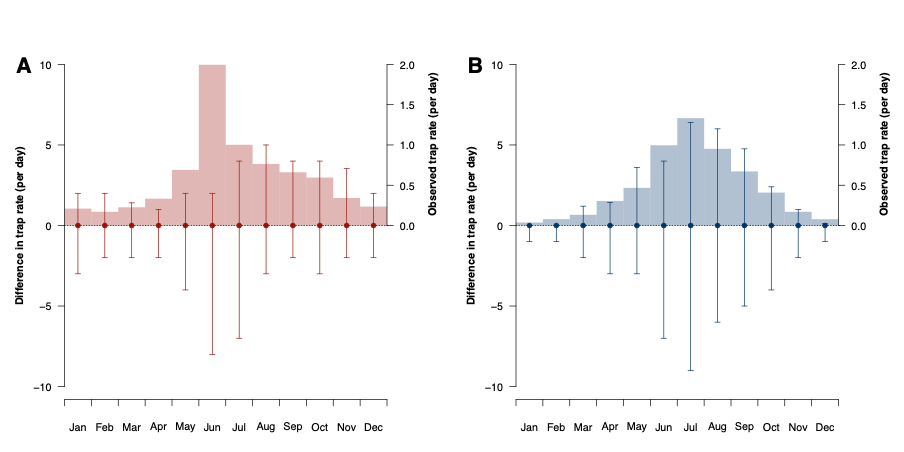

Supplement: S6 Fig — Temporal variation in model predictions in abundance of Aedes aegypti (A) and Aedes albopictus (B). (PNG) [file pntd.0009063.s017.png]

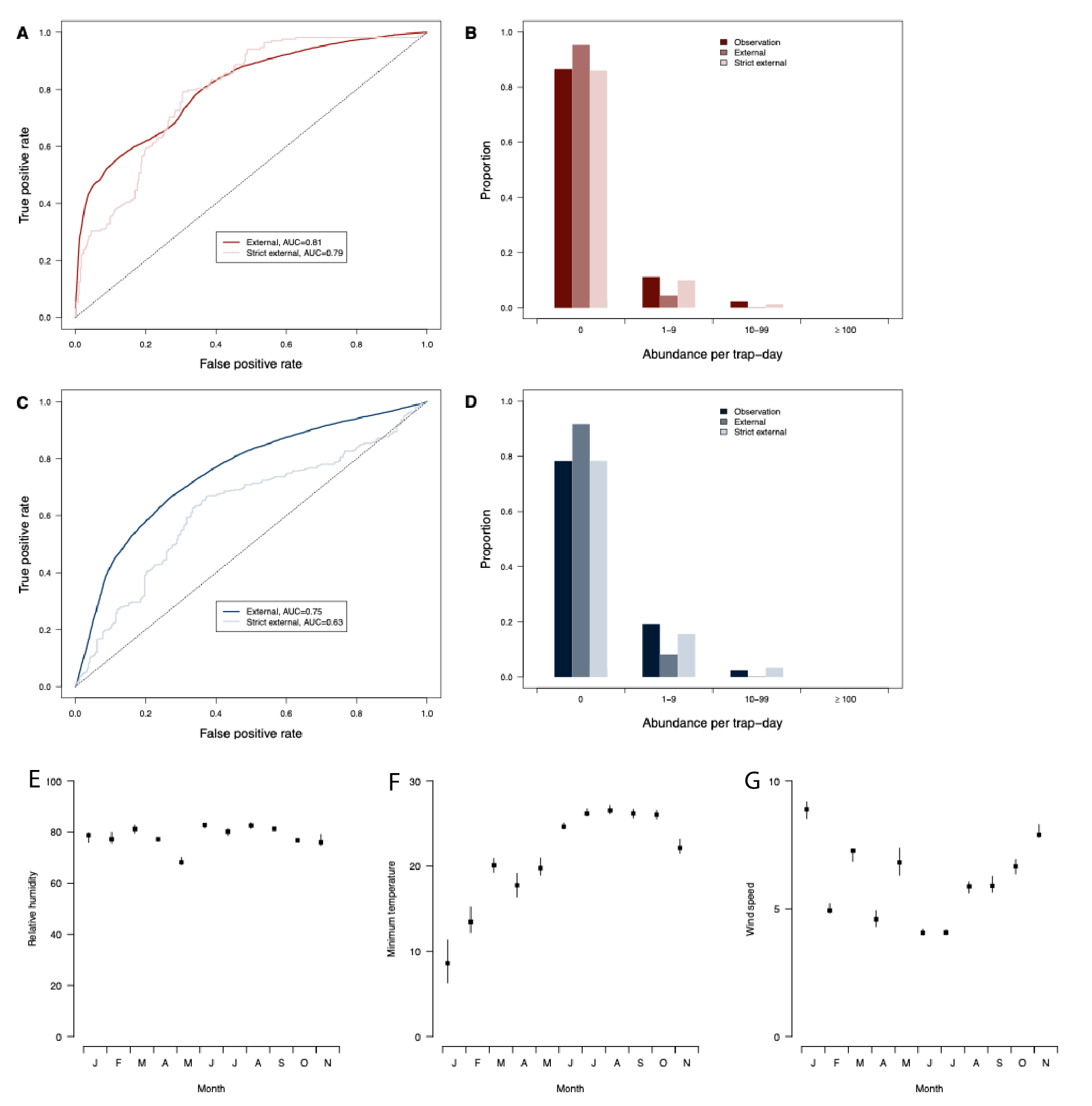

Supplement: S7 Fig — Model performances on predicting occurrence (A and C) and abundance (B and D) for external testing dataset. Relative humidity minimum temperature and wind speed for each month of the year are shown in E, F and G. (PNG) [file pntd.0009063.s018.png]

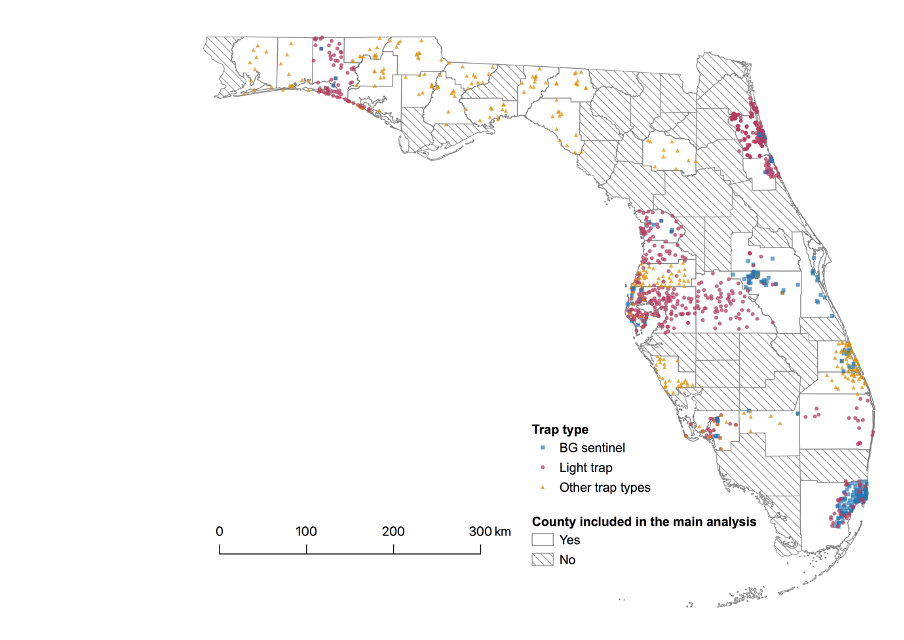

Supplement: S8 Fig — (PNG) [file pntd.0009063.s019.png]

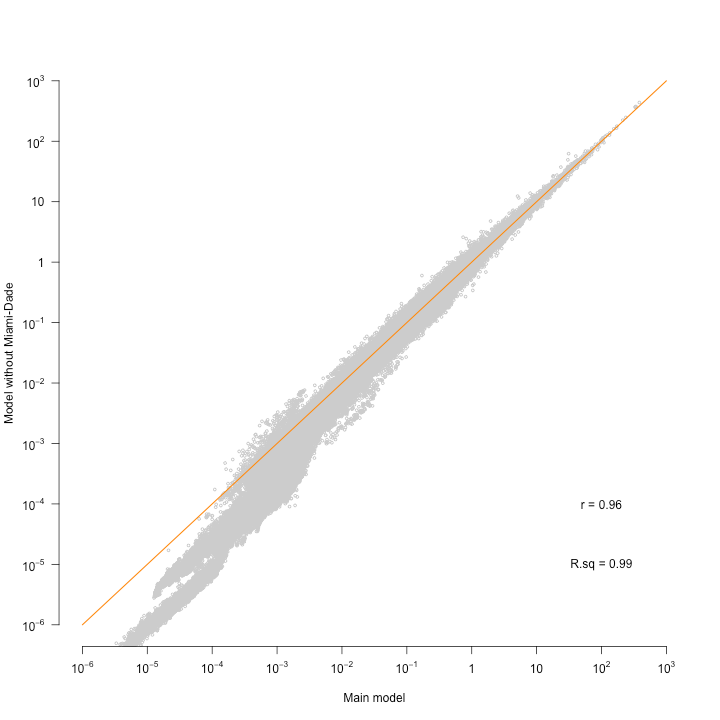

Supplement: S9 Fig — (PNG) [file pntd.0009063.s020.png]
